# Vesicle-based secretion in schistosomes: Analysis of protein and microRNA (miRNA) content of exosome-like vesicles derived from *Schistosoma mansoni*

**DOI:** 10.1038/s41598-018-21587-4

**Published:** 2018-02-19

**Authors:** Vitalie Samoil, Maude Dagenais, Vinupriya Ganapathy, Jerry Aldridge, Anastasia Glebov, Armando Jardim, Paula Ribeiro

**Affiliations:** 1 Institute of Parasitology, Macdonald Campus of McGill University, Ste-Anne-de-Bellevue, QC Canada; 2 Centre for Host-Parasite Interactions, Macdonald Campus of McGill University, Ste-Anne-de-Bellevue, QC Canada

## Abstract

Exosomes are small vesicles of endocytic origin, which are released into the extracellular environment and mediate a variety of physiological and pathological conditions. Here we show that *Schistosoma mansoni* releases exosome-like vesicles *in vitro*. Vesicles were purified from culture medium by sucrose gradient fractionation and fractions containing vesicles verified by western blot analyses and electron microscopy. Proteomic analyses of exosomal contents unveiled 130 schistosome proteins. Among these proteins are common exosomal markers such as heat shock proteins, energy-generating enzymes, cytoskeletal proteins, and others. In addition, the schistosome extracellular vesicles contain proteins of potential importance for host-parasite interaction, notably peptidases, signaling proteins, cell adhesion proteins (e.g., integrins) and previously described vaccine candidates, including glutathione-S-transferase (GST), tetraspanin (TSP-2) and calpain. *S. mansoni* exosomes also contain 143 microRNAs (miRNA), of which 25 are present at high levels, including miRNAs detected in sera of infected hosts. Quantitative PCR analysis confirmed the presence of schistosome-derived miRNAs in exosomes purified from infected mouse sera. The results provide evidence of vesicle-mediated secretion in these parasites and suggest that schistosome-derived exosomes could play important roles in host-parasite interactions and could be a useful tool in the development of vaccines and therapeutics.

## Introduction

Schistosomiasis is a major parasitic disease, affecting >200 million people in 74 countries, the majority in sub-Saharan Africa^1^. The disease is caused by blood-dwelling flukes of the genus *Schistosoma*, primarily *S. mansoni*, *S. haematobium* and *S. japonicum*. Schistosomes have a complex life cycle that requires a snail as an intermediate host. The infective stage of the parasite is a small, short-lived larva (cercaria), which is released by infected snails into fresh water and rapidly invades a human host by penetration through the skin. Shortly after penetration, the cercaria transforms into an immature parasitic larva (schistosomulum), which enters the circulation and migrates towards the hepatic portal system and mesenteries (*S. mansoni, S. japonicum*) or the venous plexus of the urinary bladder (*S. haematobium*). Larvae grow during migration, eventually maturing into adult male and female worms, which become tightly coupled and produce large numbers of eggs. The pathology associated with chronic schistosomiasis is due to the eggs. Many eggs leave the body in feces or urine, but some are trapped in tissues and induce a granulomatous immune response, leading to progressive tissue fibrosis and organ damage. Deaths due to schistosomiasis have been estimated at >200,000 per annum in Africa alone. There is no vaccine for schistosomiasis and chemotherapy relies heavily on a single drug (praziquantel), raising concerns about drug resistance^2^ which highlights the need for more research into new vaccine and drug targets.

In this regard, the secretome is of particular interest. Molecules secreted by parasites play major roles in shaping host interactions and are promising targets for vaccine or chemotherapeutic intervention. Most studies of helminth excretory/secretory products (ESP) have focused on proteins. ESP proteome analyses have been reported for a variety of nematode species^3–5^ as well as flatworms, including the liver fluke *Fasciola hepatica*^6,7^ and several life stages of *S. mansoni*, *S. japonicum* and *S. bovis*^8–14^. Parasitic worms also secrete microRNAs (miRNA)^15–19^, which could play important roles in modulating host immune responses. Secreted miRNAs are increasingly recognized as mediators of cell communication^20,21^ and their regulatory roles in the immune system are well established^22^.

Despite these advances, and the considerable efforts made to identify schistosome ESPs, very little is known about the mechanisms by which these molecules are released. Many proteins secreted by helminths are unlikely to be released through the classical secretory pathway, for example metabolic enzymes that lack a signal peptide, cytoskeletal proteins, heat shock proteins, and membrane proteins. An alternative export mechanism for these proteins is through membrane-bound vesicles^23,24^. Extracellular secretory vesicles can be formed by outward budding of the plasma membrane or formed intracellularly and subsequently released to the outside. The best characterized of these vesicles are exosomes, which are small (60–150 nm), typically cup-shaped when observed by transmission electron microscopy (TEM) and are produced from multivesicular intermediates of the endocytic pathway^24^. There is growing evidence that many proteins secreted by parasites are enclosed within exosome-like vesicles (ELVs)^19,25,26^. Recent studies of *S. japonicum*^27^ and *S. mansoni*^28,29^ suggest that schistosomes may use extracellular vesicles as a mechanism of secretion, but the molecular content of these vesicles has only partly been characterized.

Exosomes have attracted considerable interest because of their roles as mediators of intercellular communication, particularly within the immune system and in disease states such as cancer^24,30^. Exosomes are powerful agents of cell communication because of the many ways they can interact with target cells; they can bind to cell surface receptors, indirectly changing cell behavior, they can fuse with the target cell membrane, releasing their contents into the cytosol, and/or they may be taken up and internalized into phagosomes^31^. Exosomes also provide an effective mechanism for delivery of parasite molecules to a host cell, either to modulate the immune response or some other host activity that is beneficial to the parasite. Studies primarily in *Leishmania*^25,32,33^ and *H. polygyrus*^19^ have shown that parasite-derived extracellular vesicles (EVs) can be taken up by host (mammalian) cells in culture; they induce changes in gene expression in host cells and have strong immunomodulatory properties, both *in vitro* and in infected animals. *Leishmania* exosomes may be the principal mechanism by which the parasite delivers virulence factors to the host cell^25^. These studies clearly implicate EVs as important mediators of the host-parasite interaction and underscore the need for further research into their mode of action.

Here we show that *S. mansoni* release ELVs and describe the proteins and miRNAs contained within these vesicles. The results help to elucidate the mechanism by which proteins and miRNAs are secreted and provide new insight into the role of secreted products in schistosome infections.

## Results

### Purification of ELVs

**Figure 1:**
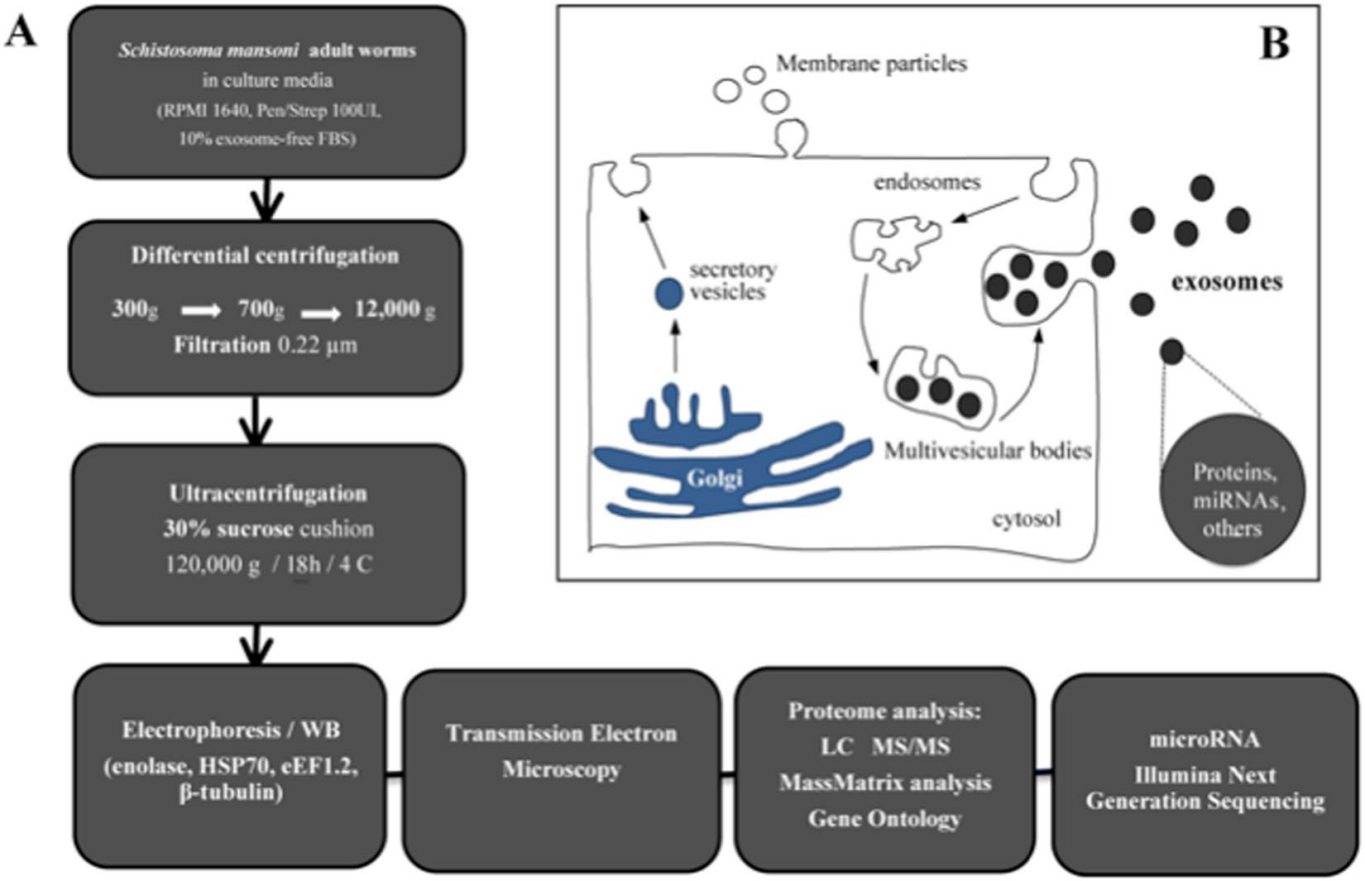
Overview of the procedure used for isolation and characterization of secreted exosome-like vesicles from *Schistosoma mansoni*. (**A**) Adult male and female worms were cultured 48-72 h in media containing exosome-depleted serum. Vesicles were purified from the culture media by differential centrifugation, followed by filtration through a 0.2 µm membrane and ultracentrifugation on a discontinuous 25, 30, 35% sucrose gradient, as described^72^. The purification was monitored by western blot (WB) analysis, using antibodies against known exosomal markers (e.g. enolase) and electron microscopy (EM), prior to analyses of protein and miRNA content. (**B**) Schematic of the two major types of secretory extracellular vesicles. Membrane particles (or ectovesicles) are formed by outward budding of the plasma membrane. Exosomes are derived from the endocytic pathway via the formation of large multivesicular body (MVB) intermediates, which fuse with the plasma membrane releasing the vesicular cargo, exosomes, as well as other contents into an extracellular environment. Alternatively, the MVBs can be directed to lysosomes and degraded [see 24 for further details].

**Figure 2:**
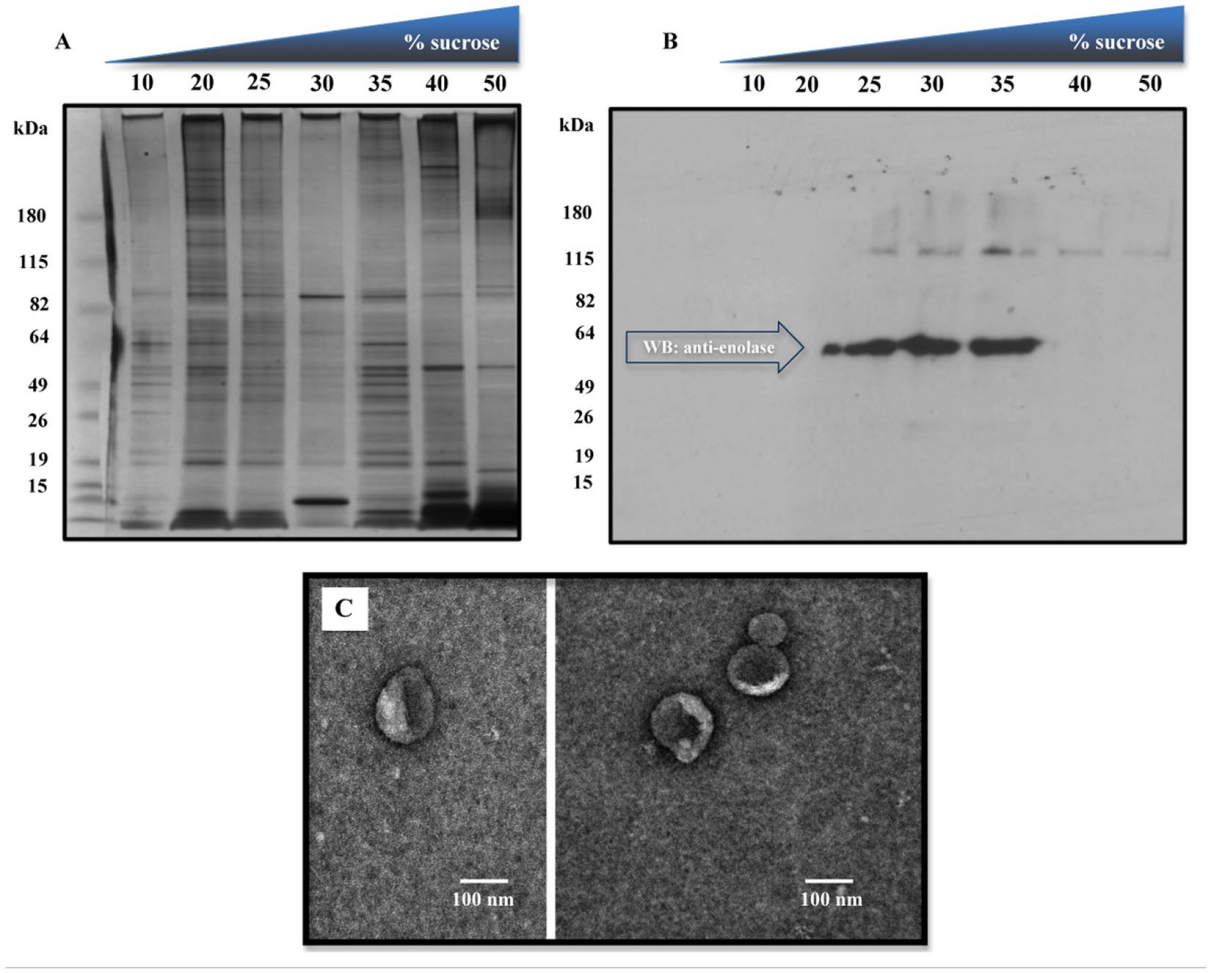
Purification of exosome-like vesicles from *S. mansoni*. Vesicles were collected from worm culture media and partially purified through differential centrifugation as shown in Fig. 1. The resulting crude vesicular pellet was resuspended in PBS, filter sterilized (0.2 µm filter) and subsequently fractionated on a discontinuous 10–50% sucrose gradient. Gradient fractions were tested for total protein content by SDS-polyacrylamide gel electrophoresis (SDS-PAGE) (**A**) and then western blotting (WB) with an antibody against enolase, a common exosomal marker (**B**). The results show enolase immunoreactivity between the 25% and 35% sucrose fractions. All subsequent purifications were performed by applying the filter-sterilized, crude vesicular pellets directly onto a discontinuous 25, 30, 35% sucrose gradient followed by ultracentrifugation, as described^72^ (**C**) Transmission electron microscopy analysis of purified exosome-like vesicles from *S. mansoni*. The scale bar indicates 100 nm.

ELVs were initially purified from culture medium by differential centrifugation, followed by filtration and sucrose density ultracentrifugation (Fig. 1) to establish the sucrose concentration required for the sedimentation and enrichment of the *S. mansoni* ELVs. Purification was monitored by Western blot analysis using an anti-human enolase antibody, a common exosomal marker, which is highly conserved with *S. mansoni* enolase (75% sequence identity). Fractions exhibited distinct protein profiles, with the 20, 25, 35 and 40% sucrose fractions containing diverse proteins, possibly corresponding to protein aggregates, smaller vesicles or plasma membrane-derived vesicles (Fig. 2A). A predominant anti-enolase reactive band was recovered from the 25%, 30% and 35% sucrose fractions (Fig. 2B), consistent with the presence of exosomes^34^. Vesicles recovered from the 30% sucrose fraction were tested with antibodies against exosomal markers that are highly conserved in *S. mansoni* (anti-HSP70, anti-tubulin, anti-elongation factor 1; sequence identities 38–98% compared to mouse homologues) and all showed immunoreactive bands of the expected size (not shown). Subsequent purifications were done by overlaying the 0.2 µm-filtered 100,000 × g pellet directly onto a 30% sucrose cushion followed by ultracentrifugation^34^. EM analysis showed that the purified cup-shaped vesicles were ~100 nm in diameter (Fig. 2C), consistent with exosomes. Our experimental analysis cannot rule out the possibility that other types of secretory vesicles of similar shape and density may be present.

### Proteomics studies

LC-MS/MS analysis was repeated on three separate preparations of purified vesicles and candidate proteins identified by searching against the *S. mansoni* NCBI genome database. These analyses identified 130 *S. mansoni* proteins, including 125 known proteins and 5 annotated as hypothetical (Table 1). Matching peptides are shown in Table S2. The detected proteins include many homologues of common exosomal markers^35^ (Table 1). We note, in particular, energy-generating enzymes often associated with exosomes (e.g., enolase, pyruvate kinase, GAPDH, phosphoglycerate kinase 1), heat shock proteins (HSP70), cytoskeletal proteins (actin, tubulin, fimbrin), 14-3-3 proteins, tetraspanin, histones, vesicular traffic proteins (e.g., Rab proteins, dynein) and translational elongation factor eEF1. Their presence in the schistosome vesicles gives further confidence that these are exosomes. Importantly, proteomics data confirmed the presence of enolase.

**Table 1:** Protein content of *S. mansoni* exosome-like vesicles.

Accession#	Annotation^a^	peptides^b^	ES protein^c^ references
***Metabolic Enzymes***			
CCD82906.1	Glycogen phosphorylase [Sm]	50	8
P16641.3	Taurocyamine kinase [Sm]	30	8, 13
ABU49845.1	Creatine kinase [Sm]	28	9
**Q27877.1**	**Enolase [Sm]** ^**d**^	**25**	8–13
**CCD76480.1**	**Pyruvate kinase [Sm]**	**22**	8, 9
CCD81281.1	Glucose-6-phosphate isomerase [Sm]	22	9
**CCD82636.1**	**Lactate dehydrogenase [Sm]**	**18**	8, 9, 11
CCD75004.1	Malate dehydrogenase [Sm]	17	8, 9, 11, 12
CCD75874.1	Phosphoenolpyruvate carboxykinase [Sm]	17	8, 9, 11, 12
XP_002571535.1	Ornithine-oxo-acid transaminase [Sm]	9	9
P41759.1	Phosphoglycerate kinase [Sm]	8	8, 9, 11–13
CCD76263.1	Transketolase [Sm]	8	9, 12
XP_002581246.1	Phosphoglucomutase [Sm]	7	8, 9
**CCD79691.1**	**Fructose 1**,**6-bisphosphate aldolase [Sm]**	**7**	8, 9, 11–13
CCD59265.1	Aldehyde dehydrogenase, putative [Sm]	4	9, 11
CCD59437.1	Adenosylhomocysteinase, putative [Sm]	4	
**CCD75628.1**	**Glyceraldehyde-3-phosphate dehydrogenase [Sm]**	**3**	8, 9, 11–13
P09383.1	Hypoxanthine-guanine phosphoribosyltransferase [Sm]	3	
AFH56663.1	Methylthioadenosine phosphorylase [Sm]	3	9
XP_002577577.1	Aconitate hydratase [Sm]	3	11
Q27778.1	6-phosphofructokinase [Sm]	3	8, 9
CCD81640.1	Long-chain-fatty-acid-CoA ligase [Sm]	2	
CCD75611.1	Glycogenin-related [Sm]	2	
CCD58348.1	6-phosphogluconate dehydrogenase,putative [Sm]	2	
***Xenobiotic/Redox Metabolism***			
P09792.1	Glutathione S-transferase 28 kDa isozyme [Sm]	14	8, 9, 11–13
XP_002582203.1	Glutathione S-transferase 26 kDa [Sm]	10	9, 11–13
CCD59704.1	Aldo-keto reductase, putative [Sm]	6	11
AAA29889.1	Glutathione S-transferase, partial [Sm]	5	9, 11
CCD77979.1	Glyoxalase I [Sm]	3	
***Proteases***			
CCD59179.1	Thimet oligopeptidase (M03 family) [Sm]	19	
CCD78710.1	Leucine aminopeptidase (M17 family) [Sm]	14	8, 9
CCD76462.1	Calpain (C02 family) [Sm]	11	9
P09841.3	Hemoglobinase (Antigen SM32) [Sm]	7	
CCD74613.1	Cathepsin B-like peptidase (C01 family) [Sm]	6	9, 10
XP_002572619.1	Prolyl oligopeptidase (S09 family) [Sm]	5	
CCD77256.1	SpAN g.p. (M12 family) [Sm]	5	
AHB79081.1	Serine protease 2 precursor [Sm]	3	10
XP_002578277.1	Subfamily M12B unassigned peptidase (M12 family)[Sm]	3	
CCD80658.1	Family S9 non-peptidase homologue (S09 family) [Sm]	3	
CCD79473.1	Xaa-Pro dipeptidase (M24 family) [Sm]	2	
***Fatty acid binding***			
1VYG	Fatty Acid Binding Protein [Sm]	3	8–11, 13
***Transporters/Channels***			
CCD60986.1	Plasma membrane calcium-transporting atpase [Sm]	14	
CCD78964.1	Sodium potassium transport ATPase alpha subunit [Sm]	8	
CCD77470.1	Glucose transport protein [Sm]	7	
CCD80392.1	Cation-transporting ATPase [Sm]	5	
XP_002578298	Choline transporter-like protein 2 (Ctl2) [Sm]	4	
CCD75891.1	Aquaporin-3 [Sm]	3	
XP_002578385.1	Chloride channel protein [Sm]	3	
CCD77770.1	Anion exchange protein [Sm]	2	
***Signal Transduction and Biological Regulation***			
**Q26540.1**	**14-3-3 protein homolog 1 [Sm]**	**10**	8, 9, 12, 13
**CCD75054.1**	**14-3-3 epsilon [Sm]**	**9**	9, 12, 13
**XP_002578585.1**	**Annexin [Sm]**	**8**	9, 10
CCD74824.1	sh3 domain grb2-like protein B1 (endophilin B1) [Sm]	6	
CCD74840.1	Kynurenine aminotransferase [Sm]	6	
CCD77507.1	Talin [Sm]	6	
XP_002577660.1	Integrin alpha-ps [Sm]	6	
CCD74661.1	Integrin beta subunit [Sm]	6	
CCD79674.1	Atp-diphosphohydrolase 1 [Sm]	5	8
XP_002578604.1	rpgr-interacting protein 1 related [Sm]	4	
CCD58613.1	Hyperpolarization activated cyclic nucleotide-gated potassium channel, putative [Sm]	4	
XP_002576557.1	N, N-dimethylarginine dimethylaminohydrolase [Sm]	3	
XP_002569666.1	ral guanine nucleotide dissociation stimulator ralgds[Sm]	3	
CCD75701.1	Proline-serine-threonine phosphatase interact protein [Sm]	3	
CCD78505.1	Lip-related protein (liprin) alpha [Sm]	3	
**XP_002581393.1**	**Tetraspanin [Sm]**	**3**	
XP_002580804.1	Serine/threonine protein kinase [Sm]	3	
CCD78813.1	rap1 [Sm]	2	
CCD58662.1	Calponin homolog, putative [Sm]	2	8, 13
CCD78057.1	Voltage-gated potassium channel [Sm]	2	
**CCD80562.1**	**Syntenin [Sm]**	**2**	
***Cytoskeletal/Structural***			
CCD58796.1	Fer-1-related [Sm]	40	
**P53471.1**	**Actin-2 [Sm]**	**19**	9, 12
CCD79944.1	Alpha tubulin [Sm]	13	9, 12
CCD79871.1	Tubulin beta chain [Sm]	12	8, 9
XP_002574516.1	Tubulin subunit beta [Sm]	12	8, 9, 12
CCD76120.1	Collagen alpha-1(V) chain [Sm]	11	
CCD60034.1	Prominin (prom) protein, putative [Sm]	10	
CCD77450.1	Rab GDP-dissociation inhibitor [Sm]	7	
**CCD82967.1**	**Actin [Sm]**	**6**	8, 9, 12, 13
CCD60380.1	Synaptotagmin, putative [Sm]	6	
AAA29882.1	Fimbrin [Sm]	5	8, 9
CCD82452.1	Signal recognition particle 68 kD protein [Sm]	5	
XP_002572341.1	Gelsolin [Sm]	4	
CCD77020.1	Intermediate filament proteins [Sm]	4	
CCD76586.1	Cytoplasmic dynein light chain [Sm]	3	8, 13
XP_002572850.1	Collagen alpha chain type IV [Sm]	2	
CCD79854.1	Microtubule-associated protein 9 [Sm]	2	
CCD82782.1	Rab-2,4,14 [Sm]	2	
CCD60258.1	Rab11, putative [Sm]	2	
CCD74879.1	Ran [Sm]	2	
***Tegumental antigens***			
CCD81232.1	200-kDa GPI-anchored surface glycoprotein [Sm]	24	
CCD76403.1	Tegumental protein Sm 20.8 [Sm]	9	8, 13
P14202.1	Tegument antigen SmA 22.6 [Sm]	7	10
CCE94318.1	Tegumental antigen [Sm]	3	
CCD59158.1	Sm23, putative [Sm]	2	
CCD76286.1	Sm29 [Sm]	2	
***Histones***			
CCD77737.1	Histone H3 [Sm]	4	8
AAG25601.1	Histone H4 [Sm]	4	8, 12
CCD75757.1	Histone H2B [Sm]	3	8, 12
***Chaperones***			
**CAZ34365.1**	**Heat shock protein 70 (hsp70)−4**, **putative [Sm]**	**7**	8, 9, 12, 13
Q26565.1	Peptidyl-prolyl cis-trans isomerase [Sm]	5	8, 11
CCD76203.1	Heat shock protein-HSP20/alpha crystallin family [Sm]	5	
XP_002577613.1	Chaperonin containing t-complex protein 1 epsilon subunit tcpe [Sm]	3	
***Translation***			
**CCD76432.1**	**Elongation factor 1-alpha (ef-1-alpha) [Sm]**	**7**	8, 9, 11, 13
**CCD79146.1**	**Eukaryotic translation elongation factor [Sm]**	**5**	9, 12
***Others***			
XP_002576729.1	SPRY domain containing protein [Sm]	10	
CCD60716.1	Cell division control protein 48 aaa family protein (transitional Endoplasmic reticulum atpase), putative [Sm]	7	
CCD82557.1	Band 4.1-like protein [Sm]	6	
CCD58670.1	Centrosomal protein of 135 kDa (Cep135 protein) [Sm]	6	
CCD82376.1	Excision repair helicase ercc-6-related [Sm]	5	
XP_002575991.1	Ubiquitin (ribosomal protein L40**)** [Sm]	4	8, 9, 11, 13
CCD76953.1	Ubiquitin-protein ligase BRE1 [Sm]	4	
XP_002578337.1	Mixed-lineage leukemia 5 mll5 [Sm]	4	
CCD58616.1	Basic helix-loop-helix transcription factor, putative [Sm]	3	
CCD77867.1	Late embryogenesis abundant protein [Sm]	3	
CCD80225.1	Zinc finger protein [Sm]	2	
***Hypothetical/unnamed***			
CCD75352.1	hypothetical protein Smp_140590 [Sm] (similar to Galectin family)	9	
XP_002570696.1	hypothetical protein [Sm] (enolase, fragment)	7	
CCD81381.1	hypothetical protein Smp_007640 [Sm] (1,6-glucosidase)	7	
CCD80386.1	unnamed protein product [Sm] (actin-like protein)	6	
CCD78834.1	hypothetical protein Smp_134750 [Sm]	5	
CCD82007.1	hypothetical protein Smp_080920.3 [Sm]	3	
CCD77946.1	hypothetical protein Smp_133590 [Sm] membrane, Ca binding vesicle fusion, neurotransmitter exocytosis	3	
CCD82509.1	hypothetical protein Smp_024220 [Sm]	2	
CCD58986.1	hypothetical protein Smp_155620 [Sm]	2	
CCD79363.1	hypothetical protein Smp_145450 [Sm]	2	
XP_002575612.1	hypothetical protein [Sm]	2	
CCD75804.1	hypothetical protein Smp_159020 [Sm]	2	
CCD76102.1	hypothetical protein Smp_006830.1 [Sm]	2	
***Host proteins*** **(** ***Mus musculus*** **)**			
XP_006536151	importin-8 isoform X3 [Mm]	4	
XP_006521203.1	keratin, type II cytoskeletal 1b isoform X1 [Mm]	3	
XP_006520948.1	keratin, type II cytoskeletal 79 isoform X1 [Mm]	3	
XP_006521177.1	keratin Kb40 isoform X2 [Mm]	2	
XP_006520575.1	keratin, type I cytoskeletal 18 isoform X1 [Mm]	2	
XP_006523578.1	axin-1 isoform X1 [Mm]	2	
XP_006531840.1	39 S ribosomal protein L21 [Mm]	2	

^a^MS data were used to search the *S. mansoni* and mouse (*Mus musculus*) genome datasets available at NCBI; Sm, *S. mansoni* protein; Mm, mouse protein. ^b^Number of unique (non-overlapping) peptides matching the designated protein across three independent experiments. ^c^Proteins previously identified in proteomics analyses of schistosome excretory/secretory (ES) products. Relevant references are provided (refer to reference list for full citations). ^d^Proteins in boldface print are featured in ExoCarta’s “top 25” list of the most common exosomal markers from all different species and tissues.

The proteins in Table 1 were compared to previously reported schistosome ES proteins. *S. mansoni* secretome studies have been done mainly on larval stages or eggs^8,11,12,14^ but a comprehensive dataset is available for adult *S. japonicum*^9^, which was used for this comparative analysis. The *S. japonicum* secretome lists approximately 100 proteins, of which about half are “atypical” (or non-secretory) proteins that lack a signal peptide^9^. We found ~50% of these atypical secretory proteins (Table 1), in particular heat shock proteins, enolase, GAPDH, GST, 14-3-3 proteins and a fatty acid binding protein. These are all present in the purified vesicles and were previously described as some of the most abundant secreted proteins in *S. japonicum* and *S. mansoni* larvae. These results suggest that a significant proportion of protein secretion in schistosomes occurs through vesicles.

**Figure 3:**
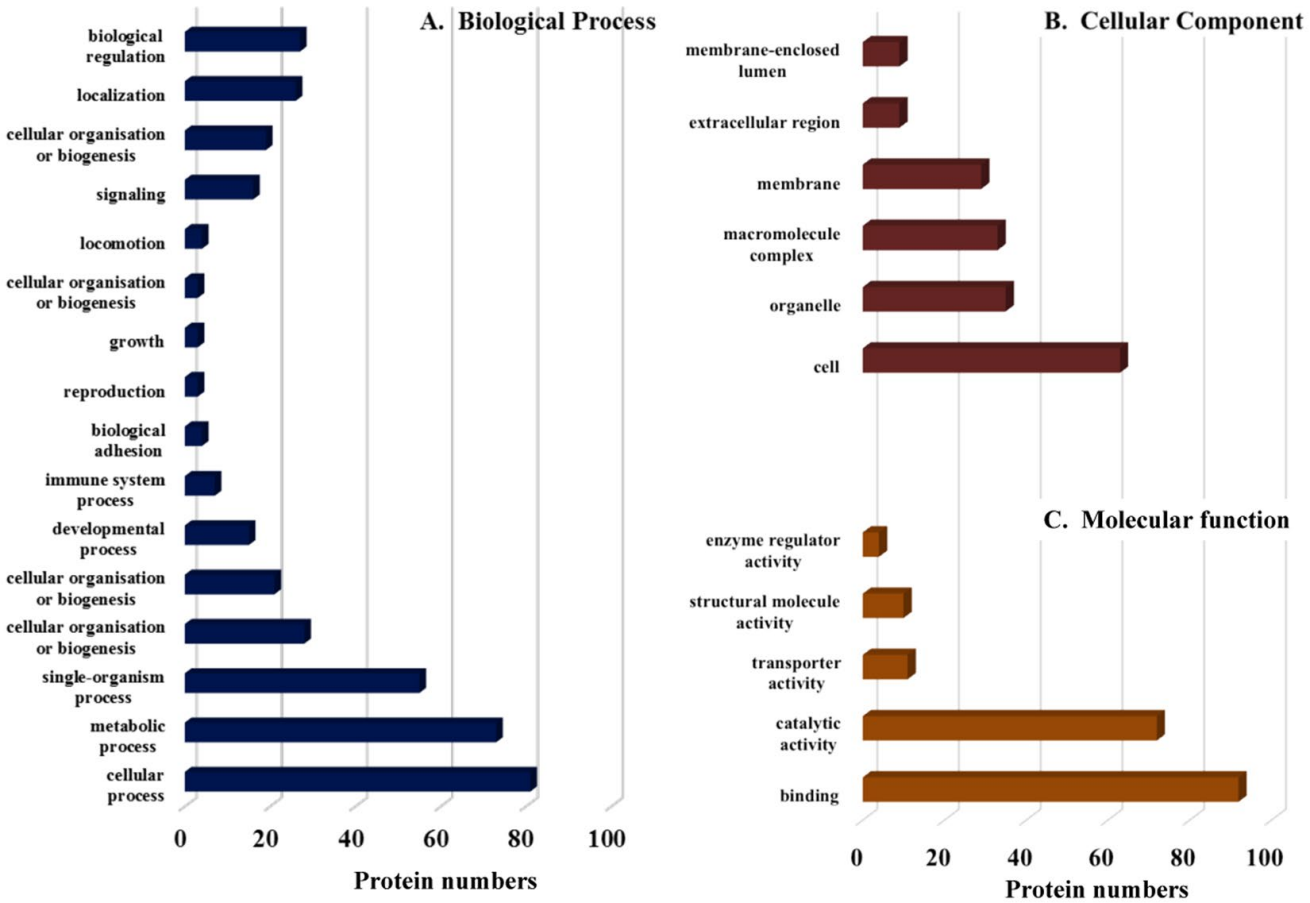
Gene Ontology (GO) analysis of proteins recovered from *S. mansoni* exosome-like vesicles. The identified proteins analyzed with Blast2GO^83^ and were classified according to Biological Process (**A**), Cellular Component (**B**) and Molecular Function (**D**), as defined by the GO consortium.

Exosomal proteins were classified by GO annotation according to molecular function, biological process and predicted cellular compartment (Fig. 3). The results show a high prevalence of proteins with catalytic and/or binding activity (“molecular function”) and proteins involved in metabolic and cellular processes (“biological process”). Enzymes are the most common type, representing nearly half of the proteins. The majority of these enzymes are involved in glucose metabolism. Enzymes from other pathways, for example taurine metabolism (taurocyamine kinase), amino acid metabolism (ornithine-oxo-acid transaminase), purine metabolism (hypoxanthine-guanine phosphoribosyltransferase, HGRPT) and detoxification (redox) metabolism (e.g. glutathione-S-transferase (GST), glyoxalase) were detected. The schistosome vesicles are rich in proteases, including metallopeptidases, cysteine peptidase and serine peptidases. The most abundant of these enzymes, based on the number of spectral matches, are thimet oligopeptidase, leucine aminopeptidase (LAP), calpain, hemoglobinase and cathepsin B.

Many proteins were assigned to GO biological processes terms that relate to signal transduction (“signalling”, “response to stimulus”, “immune processes”) and biological regulation (Table 1). Some of these proteins are common exosomal markers that have broad spectrum signalling activities, for example 14-3-3 proteins^36^. We also identified proteins associated with calcium-dependent signalling (e.g., calponin), GTP-mediated signalling (rpgr interacting protein 1), a serine/threonine protein kinase, phosphatase-associated proteins, a grb2-like protein normally involved in receptor tyrosine kinase signalling, and two putative potassium channels, one of which is activated by cAMP. A few proteins listed in this category have activities in immune processes and blood coagulation pathways, which could be important for the host-parasite interaction. One example is annexin, a calcium-dependent phospholipid-binding protein implicated in a wide range of cellular processes. Some types of annexins have powerful anti-inflammatory activity^37^; others function as plasminogen receptors and stimulate fibrinolysis, thus reducing the formation of blood clots^38^. Also noteworthy are schistosome homologues of ATP-diphosphohydrolase 1, N.N-dimethylarginine dimethylaminohydrolase (DDAH), kynurenine aminotransferase and integrins. In mammals, these proteins have important immune and/or anti-clotting effects. ATP-diphosphohydrolase 1 suppresses inflammation, inhibits platelet aggregation and prevents blood clots by breaking down extracellular pro-inflammatory and prothrombotic ATP and ADP^39^. DDAH and kynurenine aminotransferase control the levels of important regulators of immune function by indirectly controlling nitric oxide production^40^ and the levels of kynurenine, a tryptophan metabolite that regulates vasodilation and immune responses^41^. Finally, integrins mediate cell adhesion and are implicated in a wide range of signalling mechanisms associated with immunity^42^. We found alpha and beta integrin subunits, as well as talin, which anchors integrins to the cytoskeleton and mediates subsequent signalling, in schistosome exosomes.

“Cellular compartment” analysis shows a high proportion of proteins associated with membrane-bound compartments (vesicles, organelles) as well as the plasma membrane. Membrane proteins constitute roughly 30% of identified proteins (Fig. 3). Some of the most abundant proteins in the dataset are membrane proteins, notably fer-1, a homologue of vertebrate dysferlin, which has been implicated in membrane structure, fusion, and repair associated with wound healing^43^. Other membrane proteins include transport proteins (e.g., aquaporin, glucose transporter), structural proteins (e.g., prominin), signalling proteins (e.g., tetraspanin, integrins, ion channels), membrane-associated enzymes (e.g., calpain) and a variety of schistosome tegumental antigens (e.g., 200 kDa GPI-anchored glycoprotein) (Table 1).

MS spectra were also searched against the *Mus musculus* database to test if the schistosome vesicles contained host proteins, which are known to be present in parasite-derived exosomes^19,26^. Only 7 mouse proteins were reliably identified (Table 1), of which 4 were keratins, a common contaminant in proteomics studies. Three additional mouse proteins (enolase, actin and plasma membrane calcium ATPase) were disregarded because the peptides matched the schistosome homologues with higher scores. Aside from keratins, the most significant hit was an isoform of importin, a protein typically involved in nuclear transport.

### Analysis of miRNA content

The majority of miRNA reads mapped to non-miRNA sequences (mRNAs, other noncoding RNAs), repetitive sequences, or identified as “no hits” (Table S3). These sequences were removed from the datasets and not analyzed further. Of the remaining reads, we focused on those that aligned to *S. mansoni* or Platyhelminth (flatworm) miRNAs in miRbase. Reads that did not match known miRNAs were considered only if they mapped to the genome of *S. mansoni* and the extended sequences at the mapped genome positions were predicted to form hairpins.

**Figure 4:**
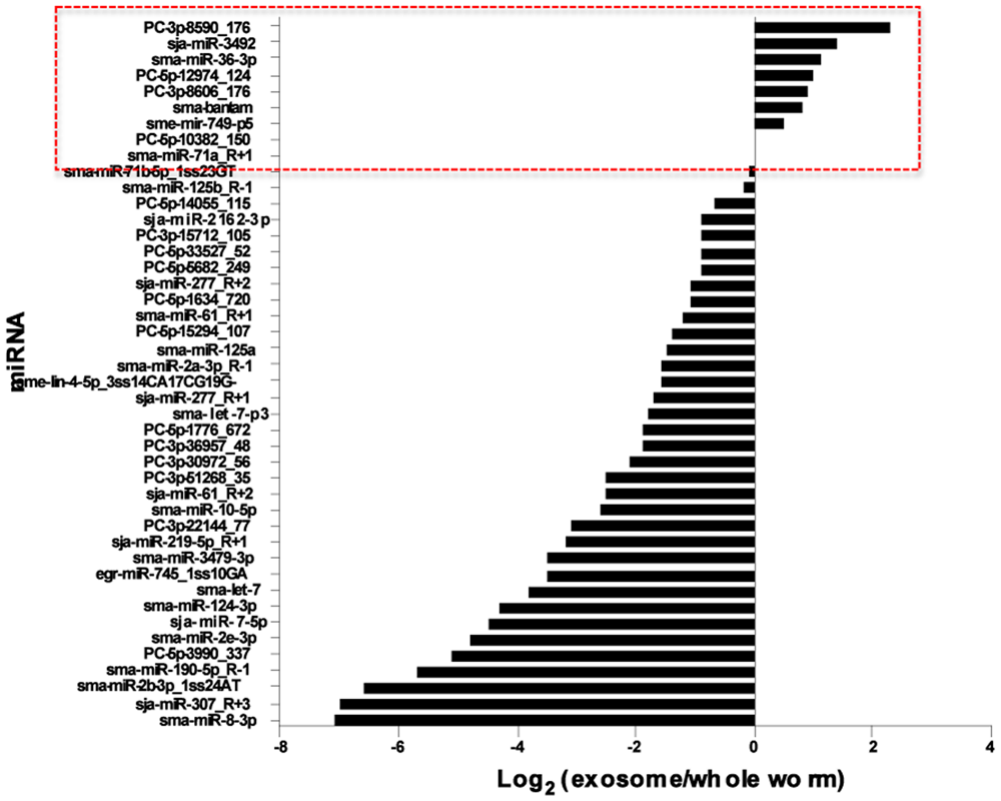
Comparative analysis of *S. mansoni* microRNAs (miRNA) obtained from whole worms and purified exosome-like vesicles. The data are shown as the Log_2_ ratio of normalized reads in the exosomal sample relative to the whole worm sample. Only the most abundant miRNAs are shown. Those miRNAs that are present at about the same level in the two samples, or are enriched in exosomes (Log_2_ ≥ 0) are marked.

The analysis identified 158 known and predicted miRNAs in whole worm extracts, 143 were also detected in exosomes but their relative abundances differed. This includes only sequences represented by >10 in one of the samples; miRNAs present at <10 reads in both samples were omitted from the analysis. The most abundant exosomal miRNAs (>100 reads) are described in Table 2 and a complete list is provided in Table S4. Among the top hits, roughly 70% are available in miRBase, including known *S. mansoni* (sma) sequences and conserved homologues from *S. japonicum* (sja) and the planarian *Schmidtea mediterranea* (sme). The remaining are novel, putative miRNAs that mapped to the *S. mansoni* genome within predicted hairpins. Most miRNAs are underrepresented in vesicles, in some instances by more than 100-fold (Fig. 4). Some, however, are present at the same or even higher levels; two of the more abundant miRNAs, sma-miR-71a and sma-miR-125b, were both present at about the same level in the two samples, and other miRNAs, such as sma-bantam and sma-miR-36-3P, were moderately enriched in vesicles. Bantam is an invertebrate-specific miRNA previously detected in serum of helminth-infected hosts and was reported to be secreted by the parasite^16,17,44^. Our results support these earlier studies and further suggest that sma-bantam is released, at least in part, in secretory vesicles.

**Table 2:** List of most abundant miRNAs in purified *S. mansoni* exosomes.

miR name^a^	miR sequence	Norm reads^b^ exosome	Norm reads whole worm
sma-miR-125b_R-1^c^	TCCCTGAGACTGATAATTGCT	43,394	49,431
**sma-bantam** ^**d**^	TGAGATCGCGATTAAAGCTGGT	**13**,**148**	**7**,**495**
**sma-miR-71a_R + 1**	TGAAAGACGATGGTAGTGAGAT	**12**,**322**	**12**,**408**
sma-miR-125a	TCCCTGAGACCCTTTGATTGCC	12,075	33,572
**sma-miR-36-3p**	CCACCGGGTAGACATTCATTCGC	**8**,**977**	**4**,**146**
sma-miR-10-5p	AACCCTGTAGACCCGAGTTTGG	3,281	19,497
sma-miR-61_R + 1	TGACTAGAAAGTGCACTCACTTC	3,145	7,471
sma-miR-2a-3p_R-1	TCACAGCCAGTATTGATGAAC	2,198	6,603
sme-lin-4-5p_	TCCCTGAGACCTTAGAGTTGT	1,893	5,902
sja-miR-2162-3p	TATTATGCAACGTTTCACTCT	1,794	3,246
sja-miR-277_R + 2	TAAATGCATTTTCTGGCCCGTT	653	1,354
**PC-5p-12974_124** ^**e**^	GAGAGATTAAGACTGAACGCC	**580**	**298**
sja-miR-277_R + 1	TAAATGCATTTTCTGGCCCGT	532	1,717
**PC-3p-8606_176**	ACGGGCTTGGCAGAATTAGCGGGG	**304**	**167**
PC-5p-1634_720	TCCCTGAGACCTTAGAGTTGTCT	275	595
**sme-mir-749-p5**	GTCCGGGGTGCAGGCTTC	**275**	**188**
sma-let-7	GGAGGTAGTTCGTTGTGTGGT	254	3,535
**sma-miR-71b-5p**	TGAAAGACTTGAGTAGTGAGACT	**243**	**254**
PC-5p-15294_107	ACACTGCGAGGCATTGAAT	207	563
**PC-3p-8590_176**	GAGATGGATAGTGGCTAGCATTT	**190**	**39**
**PC-5p-10382_150**	CCTCCGGAATCCCATAGTACT	**160**	**156**
sma-miR-3479-3p	TATTGCACTAACCTTCGCCTTG	140	1,545
PC-5p-14055_115	TGGCGCTTAGTAGAATGTCACCG	121	200
PC-5p-1776_672	TGATGGATGTAGTATAGG	117	436
**sja-miR-3492**	ATCCGTGCTGAGATTTCGTCT	**111**	**42**

^a^Only the most abundant exosomal miRNAs (>100 reads) are shown. A complete list of all the miRNAs that were detected is provided in Table S2. ^b^Normalized (“norm”) reads were calculated after adjustment for the size of the library, as described in the Methods; ^c^The suffix R followed by a negative number indicates that the read sequence is shorter than the annotated miRNA in miRbase by one (R-1) or two nucleotides (R-2) at the 3′ end. Conversely, R + 1, R + 2 show that the read sequence is longer than the annotate miRNA by one or two nucleotides; ^d^miRNAs indicated by bold-face printare present at the same or higher levels in exosomes compared to whole worms. ^e^The prefix PC (potential candidate) is used to describe novel miRNA sequences.

### Detection of exosomal miRNAs in infected mice

**Figure 5:**
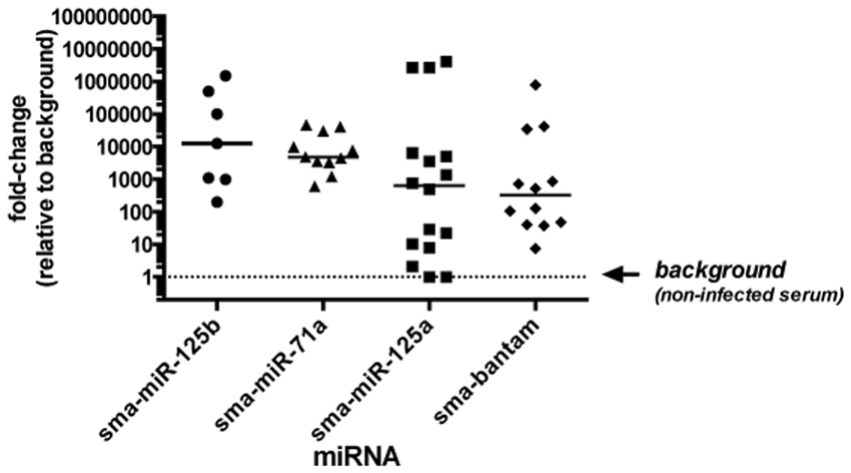
Quantitative qRT-PCR analysis of *S. mansoni* exosomal miRNAs in sera of infected mice. Circulating exosomes were purified from sera of *S. mansoni* –infected mice at 6–7 weeks post-infection or uninfected controls of the same age, using ExoQuick. RNA was extracted from the purified exosomes and then used for amplification of four *S. mansoni* miRNAs (Sma-mir-125a, Sma-mir-125b, Sma-mir-71a, Sma-bantam) by qRT-PCR. Data were median normalized relative to a “spike-in” synthetic miRNA^17,80^ and are shown as the fold-change relative to the uninfected control sample (background).

To test if *S. mansoni* releases exosome-like vesicles *in vivo*, we purified exosomes from serum of infected mice and screened for schistosome-derived miRNAs by qRT-PCR. The average worm burden was 30–40 worm pairs per mouse. As controls, we used uninfected mice maintained for the same length of time. Although most vesicles isolated using ExoQuick kit are likely of host origin, we hypothesized that even a small proportion of parasite-derived exosomes would be sufficient to detect specific *Schistosoma* miRNAs by qPCR. To facilitate detection, we focused on the abundant *S. mansoni* exosomal miRNAs from the RNA seq analysis and used a stem-loop RT method^45^ combined with TaqMan probe-based qPCR to improve specificity. Attempts to amplify miRNA using a polyA tailing RT method^46^ and SYBR green for qPCR produced non-specific results (not shown). In contrast, the stem-loop TaqMan probe method was able to specifically detect sma-miR-125a, sma-miR-125b, sma-bantam, sma- miR-71a (Fig. 5). The uninfected sample provides a measure of background amplification, presumably due to non-specific amplification of homologous sequences present in mouse-derived exosomes. The four miRNAs tested showed significant (p < 0.05) amplification compared to background levels and their median signal-to-noise ratios ranged from ~300- to 12,000-fold (Fig. 5). It was surprizing that ELVs isolated from infected mice showed notable difference in the various miRNA level. This was particularly notable for sma-miRNA-125b (Fig. 5).

## Discussion

Exosomes are important agents of intercellular communication in mammals, invertebrates and even prokaryotes^23,24^. For pathogens, exosomes constitute a powerful mechanism by which virulence factors and other bioactive molecules can be delivered to a host cell to promote infection. This has been well documented for microbial pathogens, in particular fungi, *Leishmania* and *T. cruzi*^25,47^. Evidence for exosome-mediated secretion in helminth infections is more limited^19,26,27^ and little is known about their functional roles. We further show that *S. mansoni* releases ELVs and characterize adult schistosome exosomal proteins and miRNAs.

Purified *S. mansoni*-secreted exosomes and biophysical characterization demonstrated that these vesicles were bona fide exosomes rather than crude vesicular pellets or large protein aggregates. Our approach selects for exosomes based on their distinctive size and floatation properties on a sucrose gradient^34^. Previous studies have shown that exosomes sediment at density corresponding to ~30% sucrose^34^, a finding confirmed here. This protocol minimizes contamination by larger membrane vesicles that typically have higher densities^34^. EM analysis confirmed that the ELVs used in the proteomic studies were ~60–150 nm in diameter and contained most of the “signature” (top 25) exosomal markers described in ExoCarta^35^. We recognize there may be other secretory vesicles (e.g., plasma membrane-derived ectovesicles) that have similar size and density. Thus, we refer to the material under study as exosome-like vesicles (ELVs).

A large proportion of the proteins recovered from *S. mansoni* ELVs are metabolic enzymes, particularly enzymes associated with glycolysis. The prevalence of glycolytic enzymes in exosomes has been reported in other organisms; many of the signature exosomal proteins in ExoCarta are glycolytic enzymes, such as enolase, aldolase, GAPDH, etc. For schistosomes, the discovery of these enzymes in vesicles helps to explain how they are secreted by the parasite, despite lacking classical secretion signal sequences. Why exosomes contain so many glycolytic enzymes is unclear. It is possible that exosomes increase glycolytic activity in a target cell after docking and fusion. Schistosome ELVs contain abundant glycogen phosphorylase and a glucose transporter, which would make more glucose available for metabolism, either through glycogenolysis or increased transport from the outside. Alternatively, these enzymes could have other activities. It is well known that glycolytic enzymes have “moonlighting” functions unrelated to glycolysis. For example, extracellular forms of enolase, aldolase and GAPDH bind to mammalian plasminogen^48^. If any of these enzymes is present on the surface of the schistosome vesicles, as was recently shown for *Leishmania*-derived exosomes^49^, it is tempting to speculate that the plasminogen binding activity could help prevent blood clots and facilitate parasite migration, an important activity for the blood-dwelling schistosome. Other “moonlighting” activities of extracellular glycolytic enzymes include adherence to the extracellular matrix, angiogenesis and the modulation of immune function^48^, all of which could be important for schistosomes.

Possible roles of exosomes in modulation of clotting pathways deserve consideration. Like other blood parasites, schistosomes must prevent blood clotting and remove clots once formed. This is of particular importance to *S. mansoni* because of the large size of the worm pair relative to the narrow mesenteric veins in which they reside. The presence of worms is likely to disrupt blood flow and stress the vessel wall, conditions that stimulate coagulation. Schistosomes employ a variety of mechanisms to prevent platelet aggregation and disrupt formation of clots^50^. Interestingly, some of the proteins found in the *S. mansoni* exosomes have anti-clotting activities, including ATP-diphosphohydrolase 1 (ATPDase-1), a membrane-associated protein that hydrolyzes extracellular prothrombotic ATP and ADP, inhibiting platelet aggregation and activation^39^. Previous studies of the schistosome homologue of ATPDase-1 revealed that the enzyme is present on the tegument^51^ and has ATP and ADP hydrolyzing activity^52^. Our results show that this enzyme is also secreted in vesicles. Secretion of ATPDase-1 was not reported *in vitro*^52^, but the methods employed may not have detected vesicle-associated enzyme. Exosomal ATPDase-1 may represent an important mechanism of haemostatic control. Other exosomal proteins with potential anti-clotting effects include the glycolytic enzymes listed above (enolase, GAPDH, aldolase) due to their plasminogen binding activity^48^, the schistosomal antigen Sm22.6, which inhibits thrombin^53^, annexin^38^ and the calcium-dependent protease calpain. In the host, calpain has a variety of functions, one of which is the regulation of platelet aggregation^54^. Vesicles carrying parasite calpain could alter normal calpain activity in the host and perhaps disrupt endogenous control of platelet activation. Likewise, if parasite proteins such as integrins and talin function in the same way as the mammalian homologues, vesicle-mediated delivery of these proteins to host cells could help the parasite to modulate the immune response and, importantly, control the formation of blood clots.

*S. mansoni* exosomes carry a variety of other proteases, including metalloproteases, cysteine and serine proteases that are believed to play important roles in exosome-mediated signaling^55^, contribute to invasion, migration, nutrient acquisition, immunomodulation and, haemostatic control^56^. Research on schistosome proteases has focused on secreted enzymes (e.g., cathepsin B) and cercarial elastases that contribute to skin penetration by infective larvae. There is a rich diversity of other proteases in schistosomes, most of which have only recently been identified^57^ and their functions are poorly understood. Our results show that some of these enzymes are secreted in vesicles and therefore could have important roles in the host-parasite interaction. Examples include a novel type 2 serine protease (SmSP2)^58^, a prolyl-oligopeptidase of the S9 serine protease family (SmPOP), recently shown to target bradykinin and angiotensin I^59^ and a homologue of leucine aminopeptidase (LAP). LAP is of considerable interest in flukes due to its involvement in the immune response to schistosome eggs and egg hatching^60^. LAP is present in the adult worm gut, where it contributes to digestion of blood proteins, in the parasite tegument^61^, and is secreted^8,9^, though it lacks a signal peptide. LAP secretion by *F. hepatica* is vesicle-mediated^26^ and our results suggest a similar mechanism for *S. mansoni*.

A proteomic analysis of ELVs derived from adult *S. mansoni* has been recently reported^28^. Although we identified some common proteins, notable discrepancies are evident (Table S5). Proteins found in both studies include enolase, GAPDH, glutathione-S-transferase, calpain, LAP, Sm20.8 and Sm22.6. Notable differences include glycogen phosphorylase, taurocyamine kinase and cathepsin B, high abundance proteins detected in this study that were also found in schistosome ESP^8–10,13^. Differences could be due to variations in protocols.

Proteomic analysis of schistosomulae-derived EVs revealed shared proteins between schistosomulae- and adult worm-derived EVs (e.g., taurocyamine kinase, enolase, glutathione S-transferase, calpain, 14-3-3 epsilon and Sm20.8; Table S5), but also differences. For example, schistosomulae EVs did not contain glycogen phosphorylase or glucose transporters^29^. LAP, the enzyme responsible for the final stage in catabolism of host hemoglobin^61^ was also absent from schistosomulae-derived EVs^29^, probably because the gut is not functional early in schistosomulum development^62^. Such variations in ESPs between different parasite life stages have previously been reported in helminths^63,64^ and could be used as specific life-stage markers in diagnosis.

Exosomes carry different types of RNA molecules, including miRNAs^21^. miRNAs are small molecules of ~23 nucleotides that regulate post-transcriptional silencing of genes. They bind to complementary sequences, typically in the 3′ untranslated region (3′-UTR) of a target transcript, resulting either in translational repression or sequence-specific RNA degradation^65^. miRNAs can be secreted and much of that secretion is vesicle (exosome)-mediated. Exosomal miRNAs are more stable than extravesicular forms and can be delivered to a target cell when exosomes fuse with the cell membrane^21^. Helminth parasites have a rich diversity of miRNAs^44,65–68^, some of which are secreted^15–17,19,69^, and helminth-secreted miRNAs modulate expression of specific host genes^19^, suggesting an important role in the host-parasite interaction. Here we provide evidence of vesicle-mediated miRNA secretion in adult schistosomes. Over 140 miRNAs were identified in purified vesicles, some at very high levels, including miRNAs found in sera of schistosome-infected animals^16,17^. We confirmed the presence of schistosome miRNAs in circulating exosomes from infected mice by qRT-PCR, indicating that exosomal miRNA secretion occurs in the infected host.

The biological relevance of schistosome exosomal miRNA secretion is unknown. Preliminary searches for potential human targets using computational tools (TargetScan Custom (5.2)) detected conserved seed regions in many of the schistosome miRNAs described here, suggesting these could, in principle, recognize human transcripts. For example, sma-miR125b, an abundant miRNAs in schistosome vesicles, has >600 potential human targets based on a conserved 8-mer seed region of the mature miRNA. Similar analysis of Sma-bantam, an invertebrate-specific miRNA, identified 39 potential human targets (not shown). These bioinformatics analyses must be refined and combined with gene expression studies in the context of different host cell environments to elucidate miRNA function. As previously observed in *D. immitis*^64^, it is likely that ELVs secreted by male and female schistosomes vary in content. It was also noted that the level of some miRNAs, in particular sma-miR125b, showed a broad variation in the level of miRNA found in the ELVs isolated from the serum of individual mice. These differences could be due to; i) genetic difference in the murine host since CD1 are outbreed mice which may have different responses to *S. mansoni* infections, ii) difference in worm burdens, or iii) possible differences in the stability of the miRNA transcript. It is also possible that some of the vesicular traffic is directed towards other schistosomes as a mechanism of animal-to-animal communication, as suggested recently for *C. elegans*^70^. Future studies will need to consider potential miRNA targets in the parasite as well as the host. miRNAs found in schistosomulae EVs^29^ were not among the 25 most abundant miRNAs in adult ELVs (Table 2), suggesting that the presence of these miRNA in schistosome ELVs may be stage-specific.

It is easy to envision that vesicles secreted within the confines of the mesenteric veins could directly target the endothelial lining of the blood vessels, platelets or cells of the immune system. Most research on parasite-derived vesicles has focused on the immune response. Protozoal pathogen exosomes have either pro- or anti-inflammatory effects, depending on the parasite and type of vesicle^25^, and modulate expression of a variety of host genes associated with immunity^71^. Whether these effects extend to schistosomes is unclear. A recent study of *S. japonicum* reported that treatment of macrophage RAW264.7 cells with parasite-derived vesicles promoted M1-type polarization and increased production of pro-inflammatory cytokines such as TNF-α and IL-12^27^. We tested *S. mansoni* exosomes in cultures of bone marrow-derived mouse (C57/BL6) macrophages and detected no change in TNF-α or IL12 production at concentrations of up to 50 µg exosomal protein/ml (data not shown). This discrepancy could be a function of the different target cells (RAW264.7 versus bone-marrow derived primary cells), different purity of vesicle preparations or different species of schistosome.

We have characterized miRNAs present in adult *s. mansoni* ELVs, which constitute the first report of exosomal miRNA secretion from adult schistosomes. We also provide a molecular profiling of exosomal protein content, offering contrast to a previous report^28^ (Table S5), which is possibly due to differences between our protocols. Both proteomic and miRNA analysis of schistosomulae-derived EVs^29^ revealed variations between schistosomulae- and adult worm-derived EVs (Table S5), which could serve as specific life-stage markers in diagnosis. However, further investigation will be required to exploit their potential in the development of vaccines, therapeutics and diagnostic methods.

## Methods

### Parasites

*Schistosoma mansoni* adults were obtained from CD1 mice after a six week experimental infection by perfusion^72^. Mice were maintained in the animal facility at the Small Animal Research Unit (SARU) on the Macdonald Campus of McGill University (Montreal, Canada) according to the McGill University Animal Care Committee (Permit # 2001–3346). Briefly, mice were euthanized by CO_2_ asphyxiation and cervical dislocation and *Schistosoma mansoni* adults harvested from mesenteric venules and thoroughly washed with Phosphate-Buffered Saline (PBS), and maintained in RPMI-1640 medium supplemented with 100 U penicillin, 100 mg/mL streptomycin, and 10% exosome-depleted Fetal Bovine Serum. Exosome-depletion was performed in advance by ultracentrifugation of FBS under sterile and apyrogenic conditions for 18 h at 120,000 × g and 4 °C as described^73^. FBS was of the highest grade and lowest endotoxin level (Life Technologies; Ref: 16000). Adult male and female worm pairs were maintained in 6-well plates for 72 h at a density of 20 worms (10 males and 10 females)/well in 10 ml culture medium at 37 °C. Conditioned medium was collected after 48 and 72 h. Worms remained active during this period of incubation, with no apparent change in morphology or movement. All experiments and protocols were approved by the McGill University Animal Care Committee according to the high standards established by the Canadian Council on Animal Care.

### Extracellular vesicle isolation

ELVs were purified by differential centrifugation followed by membrane filtration and sucrose density ultracentrifugation as described^34,73^. Briefly, after 48 and 72 h in culture, the parasite conditioned culture medium was collected and centrifuged first at low speed (300 × g/ 20 min), then at 3,000 × g (20 min) to remove larger debris, and the supernatant was centrifuged at 12,000 × g for 45 min. Supernatants were filter-sterilized using a 0.22 µm hydrophilic PVDF Durapore membrane (EMD Millipore; SVGV01015) and centrifuged at 100,000 × g for 2 h using a SW-41 rotor on a Beckman-Coulter ultracentrifuge. The pellet was re-suspended in sterile cell culture grade PBS (Life Technologies) and fractionated on a discontinuous 10–50% sucrose gradient (1.03–1.23 g/cm^3^), using a SW-28 rotor at 120,000 × g for 18 h. Alternatively, exosomes were purified by loading the re-suspended pellet onto a sucrose step gradient (25%, 30% and 35%)^34^, then centrifuged at 120,000 × g for 18 h as above. Following centrifugation, the 30% sucrose fraction was collected, diluted 4-5 fold with sterile PBS and vesicles were re-pelleted by centrifugation in an SW-28 rotor at 100,000 × g for 2 h. The pellets were washed once by re-suspension in PBS and centrifugation repeated at 100,000 × g. For the last spin, the pellets were re-suspended in 1.0 ml PBS, transferred to 1.5 ml Beckman-Coulter ultracentrifuge tube and centrifuged in an Optima TL100 tabletop ultracentrifuge (Beckman-Coulter) for 1.5 h at 100,000 × g using a TLA-100.3 rotor. Exosomes were quantified based on protein concentration using a Pierce BCA Protein Assay Kit (ThermoFischer Scientific). The pelleted material was snap-frozen and stored at −80 °C.

### Western blot assays

Purified ELVs (30 μg total protein) were treated with lysis buffer (Invitrogen) supplemented with 50 mM Tris (2-carboxyethyl)phosphine (TCEP) and a cocktail of protease inhibitors (Sigma-Aldrich) for 15 min at room temperature, followed by heating for 15 min at 65 °C. Proteins were resolved on a 4–20% Tris–glycine gel (Novex, San Diego, CA) and transferred to a nitrocellulose membrane according to standard protocols. Proteins were detected using goat anti-rabbit enolase antibodies (Santa Cruz Biotechnology; sc-7455). Secondary antibodies were a goat anti-rabbit IgG HRP-conjugate from Millipore (EMD Millipore).

### Electron microscopy (EM)

EM analyses of *S. mansoni* ELVs were performed at the Facility for Electron Microscopy Research of McGill University using standard protocols^34^. Briefly, exosomes were fixed in 2% paraformaldehyde in PBS for 4 h, deposited on Formvar-carbon coated EM grids, washed in PBS, treated with 1% glutaraldehyde and then washed with water. Samples were stained with a solution of 4% uranyl oxalate, pH 7, then contrasted and embedded in a mixture of 2% methyl cellulose/4% uranyl acetate, pH 4 (9:1 v/v, methyl cellulose-UA) and finally placed in methyl cellulose-UA for 10 min on ice to increase the contrast. Grids were observed in a FEI Tecnai 12 transmission electron microscope operated at 80 kV. Images were acquired with a digital AMT XR80C CCD Camera System.

### Proteomics analysis

For protein identification purified ELVs (200 μg total protein) were dissolved in a MS-compatible protein solubilizer (Invitrosol LC/MS Protein Solubilizer, Invitrogen) according to the manufacturer’s protocol and diluted with 5 volumes 50 mM ammonium bicarbonate, pH 8.5. Samples were heated to 60 °C for 30 min and sequentially reduced with 5 mM DTT (60 °C/30 min), alkylated with 5 mM iodoacetamide (20 °C in the dark/30 min) and finally treated with a small amount of DTT (10 µl 10 mM DTT) at 20 °C for 30 min to neutralize iodoacetamide. Proteins were quantified with a Pierce BCA Protein Assay Kit and samples were treated with Promega Gold Mass Spectrometry Grade Trypsin (1:50 enzyme:substrate ratio) and incubated for 14–16 h at 37 °C with shaking. Digestions were stopped with 1% trifluoroacetic acid (TFA) (Sigma-Aldrich) and peptides were purified with a C_18_ Zip-Tip (Millipore) and the peptide eluate was dried under vacuum. Peptides were reconstituted in 10 µl 0.1% formic acid before analyzing by LC-MS/MS. For analysis, peptides were fractionated on a 360 *μ*m ID x 4 mm C_18_ trap column prior to separation on a 150 µm ID x 10 cm nano-LC column (Jupiter C_18_, 3 μm, 300 Å, Phenomex) and sequenced using an ESI-LC-MS/MS system, LTQ-Orbitrap Elite hybrid mass spectrometer with a nanoelectrospray ion source (ThermoFisher, San Jose, CA) coupled with an Eksigent nano-LC 2D pump (Dublin, CA). Peptides were separated using a linear gradient of 5–40% acetonitrile (0.2% formic acid) for 53 min and a flow rate of 600 nl/min. MS survey scans were acquired in profile mode at a resolution of 60,000 at *m*/*z* 400. MS/MS spectra were acquired in the ion trap using collision-induced dissociation (CID) for multiply charged ions exceeding a threshold of 10,000 counts. Three independent LC-MS/MS experiments were performed.

### Protein data analyses

MS data were acquired using Xcalibur (v. 2.0 SR1). Peak lists were then generated with Mascot distiller (v. 2.1.1, Matrix Science) and MS processing was achieved using the LCQ_plus_zoom script. Database searches were performed against the *S. mansoni* proteome dataset available at the National Center for Biotechnology Information (NCBI) and the mouse proteome (*Mus musculus*) to test for the presence of host proteins, using MassMatrix (v. 2.4.2). The following search parameters were consistently applied: trypsin digestion with two missed cleavages allowed, cysteine carbamidomethylation as a fixed modification, methionine oxidation as variable modification, 0.8 Da tolerance for fragment ion masses, 10 parts per million mass tolerance for precursor ion masses and a minimum peptide length of 6 amino acids. A decoy reversed sequence database was included in every search. Peptide matches were considered significant if they exceeded the threshold statistical scores defined by MassMatrix^74,75^ and the false discovery rate was <5% (p < 0.05), as determined by decoy database searching. Functional annotation of protein sequences was done with Blast2GO software^76^ against the entire non-redundant NCBI nr database using default parameters. Annotation was augmented with Annotation Expander (ANNEX)^77^ and InterPro database scans were performed within Blast2Go to retrieve additional GO terms associated with functional domains.

### Illumina RNA sequencing

*S. mansoni*-derived exosomes were purified from worm-conditioned culture media as described above and the RNA contained within the vesicles was isolated using a Norgen Total RNA extraction kit (Thorold, Canada), according to the manufacturer’s protocol. Total RNA was extracted from adult male and female pairs using the same kit. All subsequent steps were performed at LC Sciences, LLC (Houston, Texas, USA) using their standard protocols. Briefly, *S. mansoni* ELV (1000 worm pairs – 20 ng of miRNA) and whole worm (400 worm pairs – 100 ng of miRNA) small RNA libraries were generated, reverse-transcribed (RT) and the resulting cDNA constructs were purified using an Illumina Truseq™ Small RNA Preparation kit. ELV and whole worm cDNA libraries were used for cluster generation on a Cluster Station fluidics device and then sequenced on an Illumina GAIIx instrument, according to vendor’s instructions. Raw sequencing reads (40 nt) were obtained using Illumina’s Sequencing Control Studio software version 2.8 (SCS v2.8), following real-time sequencing image analysis and base-calling by Illumina’s Real-Time Analysis version 1.8.70 (RTA v1.8.70). A proprietary pipeline script, ACGT101-miR v4.2 (LC Sciences), was used to analyze the data. Starting with raw sequencing reads, a series of digital filters (LC Sciences) were employed to remove reads <15 nt and unmappable sequences (low quality reads, adaptors and other “impurities” due to sample preparation and sequencing chemistry). The remaining sequences with lengths between 15 and 32 nt were mapped to *S. mansoni* and related Platyhelminth (flatworm) miRNAs available in the latest release of miRBase (v.20) (http://mirbase.org) (Accessed September 18, 2015). Mapping was also performed against other small RNAs in the RFam database (http://rfam.janelia.org), against repetitive sequences in RepBase (http://www.girinst.org/repbase) and the latest assemblies of the *S. mansoni* genome and transcriptome available at the *S. mansoni* GeneDB^78,79^ (Accessed September 28, 2015). For comparisons between ELV and whole worm samples, the number of read counts from each sample was tracked during mapping and normalized to account for differences in library size. Normalization was achieved by dividing the counts by a scaling factor that reflected the size of each library. Scaling factors were calculated according to a median count method^80^ as outlined by LC Sciences.

### Isolation of ELV RNA from mouse serum

Serum was collected from individual *S. mansoni-*infected mice 6–7 weeks post-infection and from age-matched uninfected controls. Exosomes were isolated with an ExoQuick Exosome Precipitation kit (SBI System Biosciences), starting with 500 µl serum per purification according to the recommendations of the manufacturer. The resulting exosomal pellets were resuspended in sterile lysis buffer and the RNA contained within the vesicles was isolated by column purification as specified in the kit protocol. A constant amount (10^11^ copies) of a spike-in synthetic RNA oligonucleotide (5′- CGUAUCGAGUGAUGUCACGUA- 3′) was added at the time of exosomal lysis, prior to RNA purification, and then carried through all additional steps. The spike-in RNA does not match any known miRNA in miRBase and was used for normalization of qRT-PCR data as described^17^.

#### Taqman qRT-PCR

Five highly abundant schistosome-derived miRNA sequences (Sma-Bantam, Sma-miR-71a, Sma-miR-36-3p, Sma-miR-125a and Sma-miR-125b) were selected for PCR amplification. At least 4 mismatches distinguished these miRNAs from the closest vertebrate miRNA homologue identified in miRBase, as determined by BLASTn alignments. A stem-loop method of miRNA amplification^81^ was used throughout the study. Target-specific stem-loop RT primers were synthesized and re-folded as described^45^. RT was performed according to standard protocols in a 20 µl reaction containing 0.5 mM dNTPs, 200 units MMLV reverse transcriptase (Life technologies) (or no enzyme in the -RT control tubes), 1 × First strand RT buffer, 10 mM DTT, 20 units RNase out inhibitor, 5 nM RT stem-loop primer and either synthetic miRNA or serum-derived exosomal RNA as the template. qPCR amplifications were performed in a total volume of 20 µl containing Luminaris Probe qPCR colorless master mix (Thermo Scientific), 2 µl fresh RT product, 1.5 µM sequence-specific forward primer, 0.7 µM universal reverse primer and 0.8 µM hydrolysis TaqMan probe. Primer and probe concentrations are considered optimal for stem-loop qRT-PCR of miRNAs^45^. Reactions were performed in replicates of 2–4 in an Applied Biosystems 7500 Fast thermocycler (Life Technologies) according to the following cycling protocol: 2 min at 50 °C, 10 min at 95 °C, followed by 60 cycles of denaturation at 95 °C/15 s, annealing at 58 °C/45 s and extension at 70 °C/30 s. Quantitative qRT-PCR was first performed with synthetic miRNAs to optimize reaction conditions. Amplification efficiencies were calculated from the slopes of serial dilution curves (where Efficiency (*E*) = 10 ^(−1/slope)^) and were determined to be approximately 90–100% for all five targets and the spike-in control. For qRT-PCR of serum-derived exosomal samples, we used a fixed volume (5 µl) of RNA corresponding to approximately 100 µl serum and RT was performed with two different stem-loop primers added simultaneously and at the same concentration (5 nM), one targeting the specific miRNA of interest and the other targeting the spike-in RNA used for data normalization. RT reactions were split in half and used for parallel qPCR amplification of the target miRNA and spike-in control, using appropriate primers. A complete list of all the primers and TaqMan probes used in these experiments are described in Table S1. Synthetic miRNA sequences and oligonucleotides (stem-loop RT primers, forward primers, universal reverse primer) and hydrolysis TaqMan probes were purchased from Life Technologies (Foster City, USA).

#### qPCR data analysis

A relative ΔCt method was used to compare qPCR data from schistosome-infected mice and uninfected controls. The method was adapted for the analysis of circulating miRNAs from serum and other biological fluids, where conventional data normalization based on a housekeeping gene is not feasible. Ct values were first “median normalized” to the spike-in oligo as described^17,82^ to correct for sample-to-sample variation and differences in RT efficiency. The normalized Ct (Ctn) values were then used to calculate the ΔCtn for each sample. This was done by subtracting a median Ctn of all the uninfected control samples (background amplification) from the Ctn of the test (infected) sample. Fold-change relative to the uninfected control was calculated from the formula 2^−∆Ctn^. The median Ctn values for the uninfected controls varied between 53 and 60 for the four miRNA targets. Samples where there was no detectable amplification of the target miRNA were arbitrarily assigned a maximum Ct value of 60 for data calculations.

## Electronic supplementary material

Supplementary Information
