# Supplementary material for: Vesicle-based secretion in schistosomes: Analysis of protein and microRNA (miRNA) content of exosome-like vesicles derived from Schistosoma mansoni
Source: Sci Rep. 2018 Feb 19;8:3286. doi: 10.1038/s41598-018-21587-4 (PMC5818524; doi:10.1038/s41598-018-21587-4)
Supplement: Supplementary file 1 — Supplementary Information [file 41598_2018_21587_MOESM1_ESM.docx]

**Vesicle-based secretion in schistosomes: Analysis of protein and microRNA (miRNA) content of exosome-like vesicles derived from *Schistosoma mansoni***

Vitalie Samoil^1^, Maude Dagenais^1^, Vinupriya Ganapathy^1^, Jerry Aldridge^1^, Anastasia Glebov^1^, Armando Jardim^1^* and ^a†^Paula Ribeiro^1^

^1^Institute of Parasitology and ^2^Centre for Host-Parasite Interactions, Macdonald Campus of McGill University, Ste-Anne-de-Bellevue QC, Canada

*****Corresponding author: Armando Jardim, Institute of Parasitology, Macdonald Campus, McGill University, 21,111 Lakeshore Road, Ste. Anne-de-Bellevue, Quebec, Canada, H9X 3V9,

Tel: 514-398-7727; E-mail: [armando.jardim@mcgill.ca](mailto:armando.jardim@mcgill.ca)

†deceased

**^a^** This work is dedicated to the memory of our dearly departed friend and colleague, Professor Paula Ribeiro who supervised the majority of the work reported in this manuscript.

**Table S1: List of primers, TaqMan probes and synthetic RNAs used in this study**

| **Stemloop Primers for Reverse Transcription** | |
| --- | --- |
| sma-miR- Bantam | GTC GTA TCC AGT GCA GGG TCC GAG GTA TTC GCA CTG GAT ACG ACA CCA GC |
| sma-miR- 71a | GTC GTA TCC AGT GCA GGG TCC GAG GTA TTC GCA CTG GAT ACG ACT CTC AC |
| Sma-miR-125a | GTC GTA TCC AGT GCA GGG TCC GAG GTA TTC GCA CTG GAT ACG ACG GCA AT |
| Sma-miR-125a | GTC GTA TCC AGT GCA GGG TCC GAG GTA TTC GCA CTG GAT ACG ACA GCA AT |
| Spike-in control | GTC GTA TCC AGT GCA GGG TCC GAG GTA TTC GCA CTG GAT ACG ACT ACG TG |
| **Universal Stemloop Reverse Primer for qPCR** | |
| Universal reverse primer | CCA GTG CAG GGT CCG AGG TA |
| **Stemloop Forward Primers for qPCR** | |
| sma-miR- Bantam | CAG GCG TGA GAT CGC GAT TA |
| sma-miR-71a | CAG GCG TGA AAG ACG ATG G |
| Sma-miR-125a | CAT GCA TCC CTG AGA CCC T |
| sma-miR-125b | CAC GCA TCC CTG AGA CTG A |
| Spike-in control | CAC GCA CGT ATC GAG TGA TG |
| **TaqMan Probes for qPCR^a^** | |
| sma-miR- Bantam | 6FAM-TGG ATA CGA CAC CAG CTT-MGBNFQ |
| sma-miR-71a | 6FAM- CTG GAT ACG ACT CTC ACT A-MGBNFQ |
| Sma-miR-125a | 6FAM-GAT ACG ACG GCA ATC AA -MGBNFQ |
| sma-miR-125b | 6FAM- TGG ATA CGA CAG CAA TTA-MGBNFQ |
| Spike-in control | 6FAM-CAC TGG ATA CGA CTA CGT GA -MGBNFQ |
| **Synthetic microRNAs** | |
| sma-miR- Bantam | UGA GAU CGC GAU UAA AGC UGG U |
| sma-miR-71a | UGA AAG ACG AUG GUA GUG AGA |
| sma-miR-125a | UCC CUG AGA CCC UUU GAU UGC C |
| sma-miR-125b | UCC CUG AGA CUG AUA AUU GCU |
| Spike-in control | CGU AUC GAG UGA UGU CAC GUA |

^a^ TaqMan probes were purchased with a FAM (6-carboxyfluorescein) dye label on the 5’end and a minor groove binder (MGB) and non-fluorescent quencher (NFQ) on the 3’ end.

| **Table S2: Mass spectrometry data: Protein and peptide list** | | | | | | | |
| --- | --- | --- | --- | --- | --- | --- | --- |
| **Protein Score** | **Decoy%** | **# of Peptides Identified** | **# of Spectral Matches** | **Unique peptide** | **GI (genInfo identifier)** | **Accession**  **number** |  |
|  |  |  |  |  |  |  | **Metabolic Enzymes** |
| 1476 | 0,00% | 50 | 116 | 50 | 360045358 | CCD82906.1 | Glycogen phosphorylase [Sm] |
| 816 | 0,00% | 30 | 51 | 30 | 391358187 | P16641.3 | Taurocyamine kinase [Sm] |
| 744 | 0,00% | 29 | 48 | 28 | 156118911 | ABU49845.1 | Creatine kinase [Sm] |
| 723 | 0,00% | 31 | 77 | 25 | 3023710 | Q27877.1 | Enolase [Sm] |
| 661 | 0,00% | 25 | 61 | 22 | 353230309 | CCD76480.1 | Pyruvate kinase [Sm] |
| 442 | 0,00% | 22 | 28 | 22 | 360043735 | CCD81281.1 | Glucose-6-phosphate isomerase [Sm] |
| 499 | 0,00% | 22 | 45 | 18 | 360045088 | CCD82636.1 | Lactate dehydrogenase [Sm] |
| 430 | 0,00% | 17 | 33 | 17 | 353228833 | CCD75004.1 | Malate dehydrogenase [Sm] |
| 326 | 0,00% | 17 | 21 | 17 | 353229703 | CCD75874.1 | Phosphoenolpyruvate carboxykinase [Sm] |
| 215 | 0,00% | 9 | 11 | 9 | 256070409 | XP_002571535.1 | Ornithine-oxo-acid transaminase [Sm] |
| 172 | 0,00% | 8 | 9 | 8 | 1172460 | P41759.1 | Phosphoglycerate kinase [Sm] |
| 111 | 0,00% | 8 | 10 | 8 | 353230092 | CCD76263.1 | Transketolase [Sm] |
| 138 | 0,00% | 7 | 10 | 7 | 256090542 | XP_002581246.1 | Phosphoglucomutase [Sm] |
| 78 | 0,00% | 9 | 10 | 7 | 353232336 | CCD79691.1 | Fructose 1,6-bisphosphate aldolase [Sm] |
| 147 | 0,00% | 9 | 9 | 4 | 350645988 | CCD59265.1 | Aldehyde dehydrogenase, putative [Sm] |
| 66 | 0,00% | 4 | 4 | 4 | 350645892 | CCD59437.1 | Adenosylhomocysteinase, putative [Sm] |
| 726 | 0,00% | 23 | 53 | 3 | 353229457 | CCD75628.1 | Glyceraldehyde-3-phosphate dehydrogenase [Sm] |
| 80 | 0,00% | 3 | 4 | 3 | 123502 | P09383.1 | Hypoxanthine-guanine phosphoribosyltransferase [Sm] |
| 48 | 0,00% | 3 | 3 | 3 | 383930643 | AFH56663.1 | Methylthioadenosine phosphorylase [Sm] |
| 44 | 0,00% | 3 | 4 | 3 | 256082671 | XP_002577577.1 | Aconitate hydratase [Sm] |
| 31 | 1,82% | 3 | 3 | 3 | 3122305 | Q27778.1 | 6-phosphofructokinase [Sm] |
| 46 | 0,00% | 2 | 3 | 2 | 360044093 | CCD81640.1 | Long-chain-fatty-acid-CoA ligase [Sm] |
| 41 | 0,00% | 2 | 2 | 2 | 353229440 | CCD75611.1 | Glycogenin-related [Sm] |
| 34 | 1,82% | 2 | 4 | 2 | 350643977 | CCD58348.1 | 6-phosphogluconate dehydrogenase,putative [Sm] |
|  |  |  |  |  |  |  |  |
|  |  |  |  |  |  |  | **Xenobiotic / Redox Metabolism** |
| 577 | 0,00% | 21 | 58 | 16 | 121700 | P09792.1 | Glutathione S-transferase 28 kDa isozyme [Sm] |
| 206 | 0,00% | 10 | 15 | 10 | 256093080 | XP_002582203.1 | Glutathione S-transferase 26 kDa [Sm] |
| 95 | 0,00% | 6 | 6 | 6 | 350645579 | CCD59704.1 | Aldo-keto reductase, putative [Sm] |
| 94 | 0,00% | 5 | 7 | 5 | 161007 | AAA29889.1 | Glutathione S-transferase, partial [Sm] |
| 32 | 1,82% | 3 | 3 | 3 | 353231561 | CCD77979.1 | Glyoxalase I [Sm] |
|  |  |  |  |  |  |  |  |
|  |  |  |  |  |  |  | **Proteases** |
| 343 | 0,00% | 19 | 21 | 19 | 350646132 | CCD59179.1 | Thimet oligopeptidase (M03 family) [Sm] |
| 223 | 0,00% | 14 | 21 | 14 | 360043297 | CCD78710.1 | Leucine aminopeptidase (M17 family) [Sm] |
| 119 | 0,00% | 11 | 14 | 11 | 353230291 | CCD76462.1 | Calpain (C02 family) [Sm] |
| 182 | 0,00% | 7 | 17 | 7 | 729709 | P09841.3 | Hemoglobinase (Antigen SM32) [Sm] |
| 221 | 0,00% | 6 | 13 | 6 | 353228442 | CCD74613.1 | Cathepsin B-like peptidase (C01 family) [Sm] |
| 65 | 0,00% | 5 | 5 | 5 | 256072593 | XP_002572619.1 | Prolyl oligopeptidase (S09 family) [Sm] |
| 56 | 0,00% | 4 | 5 | 5 | 353230839 | CCD77256.1 | SpAN g.p. (M12 family) [Sm] |
| 48 | 0,00% | 3 | 3 | 3 | 564131894 | AHB79081.1 | Serine protease 2 precursor [Sm] |
| 43 | 0,00% | 2 | 8 | 3 | 256084114 | XP_002578277.1 | Subfamily M12B unassigned peptidase (M12 family) [Sm] |
| 43 | 0,00% | 3 | 14 | 3 | 353233303 | CCD80658.1 | Family S9 non-peptidase homologue (S09 family) [Sm] |
| 35 | 1,82% | 2 | 3 | 2 | 353232118 | CCD79473.1 | Xaa-Pro dipeptidase (M24 family) [Sm] |
|  |  |  |  |  |  |  |  |
|  |  |  |  |  |  |  | **Fatty acid binding** |
| 76 | 0,00% | 5 | 7 | 3 | 55670478 | 1VYG | Fatty Acid Binding Protein [Sm] |
|  |  |  |  |  |  |  |  |
|  |  |  |  |  |  |  | **Transporters/channels** |
| 395 | 0,00% | 15 | 38 | 14 | 350644272 | CCD60986.1 | Plasma membrane calcium-transporting atpase, putative [Sm] |
| 68 | 0,00% | 8 | 10 | 8 | 360043551 | CCD78964.1 | Sodium potassium transporting ATPase alpha subunit [Sm] |
| 166 | 0,00% | 7 | 18 | 7 | 353231052 | CCD77470.1 | Glucose transport protein [Sm] |
| 62 | 0,00% | 7 | 6 | 5 | 353233037 | CCD80392.1 | Cation-transporting ATPase [Sm] |
| 67 | 0,00% | 4 | 6 | 4 | 256084157 | XP_002578298 | Choline transporter-like protein 2 (Ctl2) [Sm] |
| 124 | 0,00% | 3 | 9 | 3 | 353229720 | CCD75891.1 | Aquaporin-3 [Sm] |
| 33 | 1,82% | 3 | 5 | 3 | 256084334 | XP_002578385.1 | Chloride channel protein [Sm] |
| 42 | 0,00% | 2 | 12 | 2 | 353231352 | CCD77770.1 | Anion exchange protein [Sm] |
|  |  |  |  |  |  |  |  |
|  |  |  |  |  |  |  | **Signal Transduction and Biological Regulation** |
| 209 | 0,00% | 15 | 38 | 10 | 3023193 | Q26540.1 | 14-3-3 protein homolog 1 [Sm] |
| 166 | 0,00% | 11 | 14 | 9 | 353228883 | CCD75054.1 | 14-3-3 epsilon [Sm] |
| 283 | 0,00% | 11 | 26 | 8 | 256084742 | XP_002578585.1 | Annexin [Sm] |
| 186 | 0,00% | 9 | 20 | 6 | 353228653 | CCD74824.1 | sh3 domain grb2-like protein B1 (endophilin B1) [Sm] |
| 35 | 1,82% | 6 | 6 | 6 | 353228669 | CCD74840.1 | Kynurenine aminotransferase [Sm] |
| 30 |  | 6 | 12 | 6 | 353231089 | CCD77507.1 | Talin [Sm] |
| 148 | 0,00% | 7 | 14 | 6 | 256082840 | XP_002577660.1 | Integrin alpha-ps [Sm] |
| 96 | 0,00% | 6 | 9 | 6 | 353228490 | CCD74661.1 | Integrin beta subunit [Sm] |
| 34 | 1,82% | 5 | 5 | 5 | 353232319 | CCD79674.1 | Atp-diphosphohydrolase 1 [Sm] |
| 55 | 0,00% | 4 | 9 | 4 | 256084780 | XP_002578604.1 | rpgr-interacting protein 1 related [Sm] |
| 43 | 0,00% | 4 | 6 | 4 | 350646699 | CCD58613.1 | Hyperpolarization activated cyclic nucleotide-gated potassium channel, putative [Sm] |
| 122 | 0,00% | 4 | 9 | 3 | 256080579 | XP_002576557.1 | ng, ng-dimethylarginine dimethylaminohydrolase [Sm] |
| 37 | 1,82% | 4 | 6 | 3 | 256052204 | XP_002569666.1 | ral guanine nucleotide dissociation stimulator ralgds[Sm] |
| 36 | 1,82% | 3 | 5 | 3 | 353229530 | CCD75701.1 | Proline-serine-threonine phosphatase interacting protein [Sm] |
| 33 | 1,82% | 3 | 8 | 3 | 360043093 | CCD78505.1 | Lip-related protein (liprin) alpha [Sm] |
| 93 | 0,00% | 3 | 18 | 3 | 256090851 | XP_002581393.1 | Tetraspanin [Sm] |
| 34 | 1,82% | 3 | 4 | 3 | 256089413 | XP_002580804.1 | Serine/threonine protein kinase [Sm] |
| 61 | 0,00% | 2 | 4 | 2 | 360043400 | CCD78813.1 | rap1 [Sm] |
| 37 | 1,82% | 2 | 3 | 2 | 350646635 | CCD58662.1 | Calponin homolog, putative [Sm] |
| 33 | 1,82% | 2 | 4 | 2 | 360042647 | CCD78057.1 | Voltage-gated potassium channel [Sm] |
| 30 | 3,23% | 2 | 2 | 2 | 353233207 | CCD80562.1 | Syntenin [Sm] |
|  |  |  |  |  |  |  |  |
|  |  |  |  |  |  |  | **Cytoskeletal /Structural** |
| 815 | 0,00% | 40 | 105 | 40 | 350646584 | CCD58796.1 | Fer-1-related [Sm] |
| 586 | 0,00% | 19 | 46 | 19 | 1703114 | P53471.1 | Actin-2 [Sm] |
| 233 | 0,00% | 14 | 23 | 13 | 353232589 | CCD79944.1 | Alpha tubulin [Sm] |
| 313 | 0,00% | 12 | 21 | 12 | 353232516 | CCD79871.1 | Tubulin beta chain [Sm] |
| 307 | 0,00% | 12 | 21 | 12 | 256076432 | XP_002574516.1 | Tubulin subunit beta [Sm] |
| 51 | 0,00% | 11 | 12 | 11 | 353229949 | CCD76120.1 | Collagen alpha-1(V) chain [Sm] |
| 320 | 0,00% | 12 | 21 | 10 | 350645253 | CCD60034.1 | Prominin (prom) protein, putative [Sm] |
| 81 | 0,00% | 7 | 9 | 7 | 353231032 | CCD77450.1 | Rab GDP-dissociation inhibitor [Sm] |
| 150 | 0,00% | 10 | 14 | 6 | 360045419 | CCD82967.1 | Actin [Sm] |
| 169 | 0,00% | 6 | 13 | 6 | 350644919 | CCD60380.1 | Synaptotagmin, putative [Sm] |
| 106 | 0,00% | 5 | 7 | 5 | 495668 | AAA29882.1 | Fimbrin [Sm] |
| 39 | 1,82% | 5 | 5 | 5 | 360044904 | CCD82452.1 | Signal recognition particle 68 kD protein [Sm] |
| 72 | 0,00% | 4 | 5 | 4 | 256072032 | XP_002572341.1 | Gelsolin [Sm] |
| 37 | 1,82% | 4 | 7 | 4 | 353230603 | CCD77020.1 | Intermediate filament proteins [Sm] |
| 57 | 0,00% | 4 | 10 | 3 | 353230415 | CCD76586.1 | Cytoplasmic dynein light chain [Sm] |
| 32 | 1,82% | 2 | 3 | 2 | 256073059 | XP_002572850.1 | Collagen alpha chain type IV [Sm] |
| 32 | 1,82% | 2 | 2 | 2 | 353232499 | CCD79854.1 | Microtubule-associated protein 9 [Sm] |
| 64 | 0,00% | 2 | 3 | 2 | 360045234 | CCD82782.1 | Rab-2,4,14 [Sm] |
| 49 | 0,00% | 2 | 3 | 2 | 350645028 | CCD60258.1 | Rab11, putative [Sm] |
| 37 | 1,82% | 2 | 2 | 2 | 353228708 | CCD74879.1 | Ran [Sm] |
|  |  |  |  |  |  |  |  |
|  |  |  |  |  |  |  | **Tegumental antigen** |
| 282 | 0,00% | 30 | 42 | 24 | 360043686 | CCD81232.1 | 200-kDa GPI-anchored surface glycoprotein [Sm] |
| 211 | 0,00% | 13 | 23 | 9 | 353230232 | CCD76403.1 | Tegumental protein Sm 20.8 [Sm] |
| 144 | 0,00% | 7 | 11 | 7 | 135578 | P14202.1 | Tegument antigen SmA 22.6 [Sm] |
| 56 | 0,00% | 3 | 5 | 3 | 390124514 | CCE94318.1 | Tegumental antigen [Sm] |
| 111 | 0,00% | 2 | 11 | 2 | 350646174 | CCD59158.1 | Sm23, putative [Sm] |
| 69 | 0,00% | 2 | 7 | 2 | 353230115 | CCD76286.1 | Sm29 [Sm] |
|  |  |  |  |  |  |  |  |
|  |  |  |  |  |  |  | **Histones** |
| 58 | 0,00% | 5 | 6 | 4 | 353231319 | CCD77737.1 | histone H3 [Sm] |
| 55 | 0,00% | 4 | 6 | 4 | 10953803 | AAG25601.1 | histone H4 [Sm] |
| 70 | 0,00% | 5 | 8 | 3 | 353229586 | CCD75757.1 | histone H2B [Sm] |
|  |  |  |  |  |  |  |  |
|  |  |  |  |  |  |  | **Chaperones** |
| 105 | 0,00% | 8 | 9 | 7 | 238663484 | CAZ34365.1 | Heat shock protein 70 (hsp70)-4, putative [Sm] |
| 100 | 0,00% | 7 | 12 | 5 | 27805450 | Q26565.1 | Peptidyl-prolyl cis-trans isomerase [Sm] |
| 85 | 0,00% | 5 | 5 | 5 | 353230032 | CCD76203.1 | Heat shock protein-HSP20/alpha crystallin family [Sm] |
| 46 | 0,00% | 3 | 3 | 3 | 256082744 | XP_002577613.1 | Chaperonin containing t-complex protein 1 epsilon subunit tcpe [Sm] |
|  |  |  |  |  |  |  |  |
|  |  |  |  |  |  |  | **Translation** |
| 148 | 0,00% | 7 | 9 | 7 | 353230261 | CCD76432.1 | Elongation factor 1-alpha (ef-1-alpha) [Sm] |
| 55 | 0,00% | 5 | 5 | 5 | 353231791 | CCD79146.1 | Eukaryotic translation elongation factor [Sm] |
|  |  |  |  |  |  |  |  |
|  |  |  |  |  |  |  | **Others** |
| 325 | 0,00% | 10 | 25 | 10 | 256080932 | XP_002576729.1 | SPRY domain containing protein [Sm] |
| 102 | 0,00% | 7 | 8 | 7 | 350644553 | CCD60716.1 | Cell division control protein 48 aaa family protein (transitional Endoplasmic reticulum atpase), putative [Sm] |
| 126 | 0,00% | 6 | 11 | 6 | 360045009 | CCD82557.1 | Band 4.1-like protein [Sm] |
| 50 | 0,00% | 6 | 6 | 6 | 350646643 | CCD58670.1 | Centrosomal protein of 135 kDa (Cep135 protein) [Sm] |
| 48 | 0,00% | 5 | 11 | 5 | 360044828 | CCD82376.1 | Excision repair helicase ercc-6-related [Sm] |
| 105 | 0,00% | 10 | 22 | 4 | 256079432 | XP_002575991.1 | Ubiquitin (ribosomal protein L40) **[Sm]** |
| 52 | 0,00% | 4 | 4 | 4 | 353230536 | CCD76953.1 | Ubiquitin-protein ligase BRE1 [Sm] |
| 30 | 3,23% | 5 | 6 | 4 | 256084236 | XP_002578337.1 | Mixed-lineage leukemia 5 mll5 [Sm] |
| 32 | 1,82% | 3 | 5 | 3 | 350646702 | CCD58616.1 | Basic helix-loop-helix transcription factor, putative [Sm] |
| 31 | 1,82% | 3 | 3 | 3 | 353231449 | CCD77867.1 | Late embryogenesis abundant protein [Sm] |
| 32 | 1,82% | 2 | 4 | 2 | 353232869 | CCD80225.1 | Zinc finger protein [Sm] |
| 30 |  | 1 | 1 | 1 | 1084298248 | XP_018652220.1 | Gata zinc finger domain-containing protein [Sm] |
|  |  |  |  |  |  |  |  |
|  |  |  |  |  |  |  | **Hypothetical/Unnamed** |
| 183 | 0,00% | 9 | 18 | 9 | 353229181 | CCD75352.1 | hypothetical protein Smp_140590 [Sm] |
| 227 | 0,00% | 7 | 22 | 7 | 256068121 | XP_002570696.1 | hypothetical protein [Sm] |
| 103 | 0,00% | 7 | 7 | 7 | 360043835 | CCD81381.1 | hypothetical protein Smp_007640 [Sm] |
| 436 | 0,00% | 15 | 32 | 6 | 353233031 | CCD80386.1 | unnamed protein product [Sm] |
| 45 | 0,00% | 5 | 7 | 5 | 360043421 | CCD78834.1 | hypothetical protein Smp_134750 [Sm] |
| 45 | 0,00% | 3 | 3 | 3 | 360044459 | CCD82007.1 | hypothetical protein Smp_080920.3 [Sm] |
| 37 | 1,82% | 3 | 4 | 3 | 353231528 | CCD77946.1 | hypothetical protein Smp_133590 [Sm] |
| 61 | 0,00% | 2 | 4 | 2 | 360044961 | CCD82509.1 | hypothetical protein Smp_024220 [Sm] |
| 52 | 0,00% | 2 | 4 | 2 | 350646356 | CCD58986.1 | hypothetical protein Smp_155620 [Sm] |
| 37 | 1,82% | 2 | 5 | 2 | 353232008 | CCD79363.1 | hypothetical protein Smp_145450 [Sm] |
| 35 | 1,82% | 2 | 4 | 2 | 256078659 | XP_002575612.1 | hypothetical protein [Sm] |
| 33 | 1,82% | 3 | 10 | 2 | 353229633 | CCD75804.1 | hypothetical protein Smp_159020 [Sm] |
| 33 | 1,82% | 2 | 4 | 2 | 353229931 | CCD76102.1 | hypothetical protein Smp_006830.1 [Sm] |
|  |  |  |  |  |  |  |  |
|  |  |  |  |  |  |  | **Host proteins (*Mus muscuslus*)** |
| 264 | 0,00% | 4 | 11 | 4 | 569017314 | XP_006536151 | importin-8 isoform X3 [Mm] |
| 141 | 0,00% | 6 | 3 | 3 | 568992803 | XP_006521203.1 | keratin, type II cytoskeletal 1b isoform X1 [Mm] |
| 115 | 0,00% | 4 | 3 | 3 | 568992275 | XP_006520948.1 | keratin, type II cytoskeletal 79 isoform X1 [Mm] |
| 97 | 0,00% | 2 | 4 | 2 | 568992745 | XP_006521177.1 | keratin Kb40 isoform X2 [Mm] |
| 62 | 0,00% | 2 | 3 | 2 | 568991509 | XP_006520575.1 | keratin, type I cytoskeletal 18 isoform X1 [Mm] |
| 51 | 0,00% | 2 | 3 | 2 | 568998710 | XP_006523578.1 | axin-1 isoform X1 [Mm] |
| 48 | 0,00% | 2 | 2 | 2 | 569005850 | XP_006531840.1 | 39S ribosomal protein L21 [Mm] |

**Peptides**

**Metabolic Enzymes**

**Glycogen phosphorylase [Sm]** FASYLEQHYGVTINPASLFDIQVK WPVTLLEHILPR IFPAAELSEQISTAGTEASGTGNMK VQQEVEEAYKDELR RIHEYKR HLHFDGVK AKWPNDDDRIR GIAQLENVTNLK HLHFDGVKDR TQTFKDFAELWPNKFQNK ILVDLEGLEWK SQQFYHR IYYLSLEFYMGR SFDEFPKK AKWPNDDDR FGNPWEK LKLIFLENYR VAIQLNDTHPSLAIPELLR RMSLVEEEGEKR DGFFSPENPHLFK VVNNDPVVR WVDAHPVFAMPYDTPVPGYR AAPGYHMAK DSWIVNLNEIAQLK LRQEYFLVAATLQDIIRR DFAELWPNKFQNK INDVNFLR DIWGVEPSTIK TQTFKDFAELWPNK DGWQVEEPDEWLR LRQEYFLVAATLQDIIR KVAIQLNDTHPSLAIPELLR NVATSLDFYQALAR VLYPNDNFFVGK *RWVDAHPVFAMPYDTPVPGYR LIFLENYR WPNDDDRIR SQQFYHREDPKR IHEYKR DFAELWPNK NHAENISR LINSVGK LPPPFEPAIEK VQQEVEEAYK SQQFYHREDPK TVMIGGK LIIKLINSVGK ILVDLEGLEWKK MSLVEEEGEKR WIRSQQFYHR

**Taurocyamine kinase [Sm]** LGLTELQAAHEMAEGVAK GLYGEHTESPDGTYDISNK GTHGEHTESVGGIYDLSNK LGLTELDAVTEMHSGVR NFLVWINEEDHIR ISTALHNLSGEYEGTYYPLTGMSEEDRIK IKLEQVISGALK QLVEDHFLFK LIEGINAIGK TTHGATLAHMIR GLTGEHAGTYYPLTDMKEEDRK TVEGFGFGPTLTK LVNDHFLFR LPFGDLDPTGK DFFDAVIADYHK DAGGYIDWPTGR IISMQQGGNLAAVYK GAPEGVMPVEPLTYLAK ISTALHNLSGEYEGTYYPLTGMSEEDR ALLELEVMLQEYNK SLSFTDLNTYGNLVVSTR LADAIQELSK IISMQQGGNLAAVYKR SNFGDLK IPMLASLPNFK DSAFKHPAPTFGDLSK QKNFLVWINEEDHIR HPAPTFGDLSKLPFGDLDPTGK DAGGYRDWPVGR YLTDDIVKK

**Creatine kinase [Sm]** GTHGEHTESVGGIYDLSNK LGLTELDAVTEMHSGVR NFLVWINEEDHIR ISTALHNLSGEYEGTYYPLTGMSEEDRIK IKLEQVISGALK QLVEDHFLFK LIEGINAIGK TTHGATLAHMIR GLTGEHAGTYYPLTDMKEEDRK TVEGFGFGPTLTK LVNDHFLFR LPFGDLDPTGK DFFDAVIADYHK DAGGYIDWPTGR IISMQQGGNLAAVYK GAPEGVMPVEPLTYLAK ISTALHNLSGEYEGTYYPLTGMSEEDR ALLELEVMLQEYNK SLSFTDLNTYGNLVVSTR LADAIQELSK IISMQQGGNLAAVYKR SNFGDLK IPMLASLPNFK DSAFKHPAPTFGDLSK QKNFLVWINEEDHIR HPAPTFGDLSKLPFGDLDPTGK DAGGYRDWPVGR YLTDDIVKK

**Enolase [Sm]** GLQLLEEAIK AAVPSGASTGVHEALELR YMIDLDGTENKEK IIAPALINK LTSSTNIQIVGDDLTVTNPK IEEELGTAAK AGAAEAGLPLYR LAQDSGWGVMVSHR GVLTAVSNVNK LAGHEDVIMPVPAFNVINGGSHAGNK KGVLTAVSNVNK YMIDLDGTENK LTSSTNIQIVGDDLTVTNPKR NIPVTNQAAIDKYMIDLDGTENKEK GNPTVEVDLK LAMQEFMILPTGASSFTEAMQIGTEVYHNLK NIPVTNQAAIDK NIPVTNQAAIDKYMIDLDGTENK IAGYTGK FPIVSIEDPFDQDDWETWPK YDLDFKNPHSAESTWLSPDAMANMYK NPHSAESTWLSPDAMANMYK NGKYDLDFKNPHSAESTWLSPDAMANMYK YDLDFK QMISKFPIVSIEDPFDQDDWETWPK

**Pyruvate kinase [Sm]** FNEILDVVDGIMVAR KGVNLPGAHVDLPAVSEK LNFSHGSHEYHAETIK NADAVHQIR IENHEGVQR TVEMLQNMITSGMNIAR AGEWYEDMDRR KGVNLPGAHVDLPAVSEKDK FAVEHNVDMVFASFIR NIVHVLSK EAASTLKPFPRPIGIALDTK GLYPLETVQTMHR GVHPIYYGESR GDLGIEIPAEK QIHLYR VFIAQK HEVIAR AGSGSTNTLR TGLINGSGTAEVSLEVGHK QLLGDNGAYIK GVNLPGAHVDLPAVSEK AGEWYEDMDR

**Glucose-6-phosphate isomerase [Sm]** VHFVSNIDGTHIAETLKK AVLHIALR *KVNSETVLFIIASK *ELFAAGVTGDMLNSLALHK *TFTTIETMTNANSAK KINFTEDR NLIDTQSFDQLIK VNSETVLFIIASK *FAAYFQQGDMESNGK MRDDMMEGR EMFAANPK KVPQTMK TKEEVHKELFAAGVTGDMLNSLALHK DVMPGVNAVLQHMSK SNVPIMVDGK RFSEFSQILSLPGGTILLDYSK SNVPIMVDGKDVMPGVNAVLQHMSK FSEFSQILSLPGGTILLDYSK NVSHVAK VHFVSNIDGTHIAETLK *EVDYSTGPIVWGEPGTNGQHAFYQLIHQGTR *DGKEVDYSTGPIVWGEPGTNGQHAFYQLIHQGTR

**Lactate dehydrogenase [Sm]** QVVQSAYDIIR GEVLDLQHGQQFFGR VIGTGTMLDSAR SAATLNEVITGIK KLSGFEAHR VKGEVLDLQHGQQFFGR LSGFEAHR LNLVQR LSSLNPK YSANSDIVVITAGAR LGVSANSVHGYVIGEHGDSSVAVWSNVNVAGVR KSAATLNEVITGIKW KSAATLNEVITGIK IDGGTDYK NVDIFKK SAATLNEVITGIKW NVDIFK FLLGEK

**Malate dehydrogenase [Sm]** SVSDHMHDWWLGTK VLLTGAAGQIGYSLAGMVAR VVVVGNPANTNALALMK DVIFSFPVQIK WTIVQGLK EIPVTAAINDDNWIKNEFLSAIQK GAAVIAAR NEFLSAIQK GDMFGPDQEVILHLFDLEQMVESLK NAPSIPK RGAAVIAAR KDLLNANVK EIPVTAAINDDNWIK NVVVTHIPEVAFNQIDAALMVGAMPR EQGQALDKYAK ENFSALTR NAPSIPKENFSALTR

**Phosphoenolpyruvate carboxykinase [Sm]** YLHEQVGK HGVMLGAALK RPEGIPLVLQSFDWK KGSINFDGLDVDWDENFSLPK *YLHEQVGKDLPDVIQNELNNQR DLPDVIQNELNNQR TIISHVPK AINPEAGFFGVAPGTNVK IAGCIAR VLYVIPFSMGPIGSPLSK GKFEAMPK LPFIYHVNWFR *LVELGSLHK DYLLEDIDETMK FDEHGVLR YVWPGFGQNIR WISPGDLDVEIK

**Ornithine-oxo-acid transaminase [Sm]** VLEEEHLAEHAQK GLLNAIVIR LGEIFR LAPPLVIKEDELHK YNVLFIADEIQTGLGR ATEIIHK SVVELYR TPSDGYLK TLTRSISGWSDNLR

**Phosphoglycerate kinase [Sm]** ALENPERPFLAILGGAK FHVEEEGKGVSPTGEK *NVAIHLPVDFVTADKFADDANTEIR VSHVSTGGGASLELLEGK LGDVYVNDAFGTAHR LSISDVDLK NVAIHLPVDFVTADK QIHNMHIGNSLFDAPGAEIVHK

**Transketolase [Sm]** KIDSDLEGHPTPR VIDPFTIKPIDAELLAK AADVLATENINIR LGQSQPTQLQHHLEAYR SGKPEELLAK YKPESPR SVIGSTVFYPSDAVSTER ISFTSNKTDGDLGIVR

**Phosphoglucomutase [Sm]** LSGTGSSGATLR VYVDTYESDPAK FIFNDGTR TVKEPLNIQLNR LTDAIFAQSEK *YFQENGVHGFAR *MQHGYTENFIQSILNAAVGELLNKSQPVR

**Fructose 1,6-bisphosphate aldolase [Sm]** LAENISGVILFEETLHQK AYTPQENALATVR GVVPLAGTDNETTTQGLDDLASR LQQIGVENNEENRR ENVHAAQEELLK LQQIGVENNEENR RLQQIGVENNEENR

**Aldehyde dehydrogenase, putative [Sm]** KCQESLWTVDK HSDVNRYPDFELTYSTR LFLENEESIVK KSLHSEAQGVK

**Adenosylhomocysteinase, putative [Sm]** ALENPERPFLAILGGAK FHVEEEGKGVSPTGEK VSHVSTGGGASLELLEGK LGDVYVNDAFGTAHR

**Glyceraldehyde-3-phosphate dehydrogenase [Sm]** LVSWYVFIYCYSLTYFM DPANIPWDK *DSTHGTFPGEVSTENGK

**Hypoxanthine-guanine phosphoribosyltransferase [Sm]** FLADLVDGLER LISHLDSLSTK NVLVVEDIIDTGK

**Methylthioadenosine phosphorylase [Sm]** SINVYDKK VGIIGGSGFDDPNLFKK ENTFYGSKPDSLK

**Aconitate hydratase [Sm]** LSGWTSPK VLYSHLDNPK SQFFITPGSEQIR

**6-phosphofructokinase [Sm]** AEWSSLLEELVTSNK ITVLGHVQRGGSPSAFDR TDLQLESVILNK

**Long-chain-fatty-acid-CoA ligase [Sm]** YLNVLDQIANQR LAQGEYVAPER

**Glycogenin-related [Sm]** AVFMDADTVVLR QMLDIIGSVFDHVK

**6-phosphogluconate dehydrogenase,putative [Sm]** AGQAVDDFISK VKEFIENEAK

**Xenobiotic / Redox Metabolism**

**Glutathione S-transferase 28 kDa isozyme [Sm]** KHHMMGETDEEYYSVEK LIGQAEDVEHEYHK ISFQDWPK HHMMGETDEEYYSVEK TLMKPQEEKEK WMLESLAIAR HRENLLASSPR TLMKPQEEK MTLVAAGVDYEDER YLSNRPATPF MTLVAAGVDYEDERISFQDWPK ENLLASSPR IKPTIPGGR YPEIHK GFLTGKYPEIHK VIYFDGR

**Glutathione S-transferase 26 kDa [Sm]** AEISMLEGAILDIR LLLEYLGEAYEER LTQSMAILR LYDRNDGDVWR VDFLNQLPGMLK IENLPPIK LGLDFPNLPYYIDGDVK FKLGLDFPNLPYYIDGDVK NYLNSNR IAYNKEFETLK

**Aldo-keto reductase, putative [Sm]** TPAQVLLR SIPIIGLGTWNSPPGEVGVAVK LVDDGLVK VDLLMEPWVLQIAK REDVFVTSK LWNTFFRPEHVR

**Glutathione S-transferase, partial [Sm]** AEISMLEGAVLDIR LLLEHLEETYEER AYDRNEIDAWSNDKFK LGLEFPNLPYYIDGDFK VDFLNK

**Glyoxalase I [Sm]** IIGGLWPITLK LVSLDTPGK LISIQKPIDHASAFGR

**Proteases**

**Thimet oligopeptidase (M03 family) [Sm]** DLLANLINSR KLSDVEVELEMR ILRPGGSK LSDVEVELEMR LKEAEVK ERDYSVDEVALK ERDYSVDEVALKK DYSVDEVALKK FLNNVGTK IANAGLFYSR GSMDSNNTTVNER NIDSSFSGEYR YPHYFPIMQK ALNEENTLLK YYMNMVK FVSVQK ASILGFPTHADFMLDLR NGLHLDDDKRK YFPLATVK

**Leucine aminopeptidase (M17 family) [Sm]** FSDEVSVIPFPEHPSK GVTVTVDKVDAK FSDEVSVIPFPEHPSKR NSIGSESYVADEIIIAR GHQMPAAFLVVASGLDK VVHVDYKPSNSK EFSEVNPK FGVMEADNSLLNIANAIEEGR NPFIFTIATLTGHAIR AGGIMAGMHRDK KEDYAMNK VSYELQNSGDLISDIAEISTIR DIGGSDPER GITFDTGGADVK

**Calpain (C02 family) [Sm]** ERPSDLIDQLK AIGNLPDFR LEDTLLDEDQEIEQK GPEYVGVVR ISQYEIISAK ELASLELNFK TTYWTNPQFR FGEWVEVVVDDR MEFNPYLPK KETLTPK EPDFLMAR

**Hemoglobinase (Antigen SM32) [Sm]** LFNDYNHKDWYEGVVIDYR YLHSHKR *LYVSEFQGSR YLHSHK TLDQQYK LYVSEFQGSR ETDLSHVQR

**Cathepsin B-like peptidase (C01 family) [Sm]** YGPVEAGFTVYEDFLNYK YKTPYTQDK TPYTQDKHR TPYWLIANSWNEDWGENGYFR HITGETLGGHAIR YKTPYTQDK

**Prolyl oligopeptidase (S09 family) [Sm]** IRDVDLNQFEVK KTFLDPNEIDPEGLTSLR ISPLHNVK TVVPMFLILPK *QNSFDDFQAAAEYLLNHGYTNNQK

**SpAN g.p. (M12 family) [Sm]** AEDYCKNK NNSMSSNTKLVKTDK NNSMSSNTKLVK SSIEATRSITSTMEIGNLSDK NSVIVSTR

**Serine protease 2 precursor [Sm]** YKQEAAQIDHR VHVCAGAKNK AVEPHSWPWAVR

**Subfamily M12B unassigned peptidase (M12 family) [Sm]** EADGWCRVLWGK KDSLENVNQSASFYCK YGPSCHCHGLNNCIGHGNCYCSYLPR

**Family S9 non-peptidase homologue (S09 family) [Sm]** GLSIVTPR TSFHEWSK TYLWLNLTDNLTLSEGSYHSNLELK

**Xaa-Pro dipeptidase (M24 family) [Sm]** IEDNIIVTETGHELLTDVPR IPEEATIYDGELASLEQFSK

**Fatty acid binding**

**Fatty Acid Binding Protein [Sm]** LTQTQVDPK TTVTVGDVTAIR LSESHNFDAVMSK

**Transporters/channels**

**Plasma membrane calcium-transporting atpase, putative [Sm]** TALTTGSGGADSFEISPK EASDIILTDDNFSSIVK QVVAVTGDGTNDGPALK VPSANSDAYQMK IDESSLTGESDQVR TSPQDGLHEEDFSK LLPPDNPNALPK NSDKVPSANSDAYQMK LTEAVIEPMAGEGLR ESDNPMSFLLFTK TALTTGSGGADSFEISPKELQK MVTGDNVNTAR ADVGFAMGIAGTDVAK IESEHTFFVIR

**Sodium potassium transporting ATPase alpha subunit [Sm]** NLEAVETLGSTSTICSDK STEFSNENPLETK ILEIPFNSTNK IPLEELYAR IFEADTSENQTGAR AVDALSDSYGQEWTYK VLGFCDYRLPAETYPK VDNSALTGESEPQSR

**Glucose transport protein [Sm]** ENVDTFIGELREEIEVAK DLAFGNIVVGK FTQLFTQR TFDEVAR NQPVFK QGNNEGPASESLLYPR ENVDTFIGELR

**Cation-transporting ATPase [Sm]** TLTEEQYSDFAK QSIHNFEGTFVR AVMNSSRVR ATGSSDSLLDSDNESEQHGK IIFYVK

**Choline transporter-like protein 2 (Ctl2) [Sm]** ITNDDLTVTPGFSDK SLGSNIAIEH RVIHPTNSQGQVCGR VTLEYINTK

**Aquaporin-3 [Sm]** SDFVVDVDYDDSHR KSDFVVDVDYDDSHR LTSSPLVR

**Chloride channel protein [Sm]** *NGCPLGVLTKKDILR GLSLHSLFPNITKGR *DGILNDEMFTANSNVK

**Anion exchange protein [Sm]** NLRLLVLIPSK YAEIQR

**Signal Transduction and Biological Regulation**

**14-3-3 protein homolog 1 [Sm]** DLSNTDLVHIAK YTEASGNLGNEER ATTAAENLPTTHPIR YLAEVATDDAR *SAFDSAIAELDQLQDDSYK VIHGSEMK KQIAEEYR RYTEASGNLGNEER YDDMAAAMK NLLSVAYK

**14-3-3 epsilon [Sm]** VFSAVEQTEGNR LAQAAFDDAIAK LDQLSEESYK EVVEMAEELTVEER EVLESELDR GNAEKQACAK YMAEFSVDPQR LAEQLERYDEMVDAMK MKGDYFR

**Annexin [Sm]** KDAEELYQAMK SQIIGPNGETYHPTLK RDEIPFEDVEK SQGSTLEDWIR DAEELYQAMK SVWHLQEVSHLFEK GWGTDEHR IDLHNDPKK

**sh3 domain grb2-like protein B1 (endophilin B1) [Sm]** QHVFEQSLK FSQIVEEAFGTTER LSANEQLGESMDR TSYSPDLTELMK NSNYGSPGEALKK LQETVSSEYITWLR

**Kynurenine aminotransferase [Sm]** RGAFWRGNCAEAADWAASPGR YKPINLGQGFPDILPK LHVPSNELDKNESLSYDVK NNYKNSR TCRNLADHLR QELAKEIVEREIEELNR

**Talin [Sm]** TAFELGK SLMAKQDLELAWDR TIATTCADAGRTCSQLVAK SITHSETALHGENFNERQSATR RMIQMK AVLQSCTR

**Integrin alpha-ps [Sm]** VITSVEDIPSNTLLGSSVDTTDR GVGASVNTNTFER DTYLIDQTER MTEPYPLADQIDEYPVADPR QLFMGGPHFYFEK LYAPLPGLK

**Integrin beta subunit [Sm]** YSDAIYVNR KVDLNIIR HILSLTDNFEEFR IGYQISIKAK CQLRGHLHCGECK VARPSGNLDSPEGGMDALLQVAR

**Atp-diphosphohydrolase 1 [Sm]** HLNTNR LIEDPLGSLDLFSVIR LTNSLTFEQK WIDEPFR MYGFTTDESWK

**rpgr-interacting protein 1 related [Sm]** GTFLQTFNLK TSNNPLNDSPNLK RTLTANK RTSNNPLNDSPNLKDIPK

**Hyperpolarization activated cyclic nucleotide-gated potassium channel, putative [Sm]** TKSVDNLTMFK NNFADEIMLNPK EKFKQVDEYMAYR ESITSNTSIKINK

**ng, ng-dimethylarginine dimethylaminohydrolase [Sm]** SFEEFLMSPDANIPGK QGEVNLIR QNGVDVLEMEADER

**ral guanine nucleotide dissociation stimulator ralgds[Sm]** VTAEMEYLCRQAINCLK CANKMYWYLK DARDITLPK

**Proline-serine-threonine phosphatase interacting protein [Sm]** *LGHFTKNAK *WDDSKSTINDSK *NSGSELRMSR

**Lip-related protein (liprin) alpha [Sm]** SLSNHFLTK MSALEQKQNLSNQVEQIKR STQLKQQEEKVR

**Tetraspanin [Sm]** KVFDEIQQK DGVQFTEGCIK KHITSALK

**Serine/threonine protein kinase [Sm]** *CRPIIAPAGPHCGDK NGNKCRPIIAPAGPHCGDK *IVEEVESMQRR

**rap1 [Sm]** YDPTIEDSYR SNVNEIFHDLVR

**Calponin homolog, putative [Sm]** NGQILIK *EGQGIIGLQMGSNK

**Voltage-gated potassium channel [Sm]** IGTDIPK ENIMSGTDCK

**Syntenin [Sm]** SMEILK DSLGAIGIQVR

**Cytoskeletal /Structural**

**Fer-1-related [Sm]** AEADLRPAGK AIDLQPSDSSGLADPYVEIIVGQHK AREDPNENPHLEPPK DILDDQAGAGK DYDAVGADDIIGQTDIDLENR EDPNENPHLEPPK EQLPIR ETVSEIDDALK EYSDLPDKYEALNLGK FYASIGEWDK GEPNTVELPSGWTWADDWK GLTSNEIEQGSVSDK GQDTMSTIELSLR GWLAGLDDR IEMEMELVTK IPAHEVLYHDNEDYR ISVVILGPGDEAPIMK IYPLPEDPK IYPLPEDPKEQLPIR KGQDTMSTIELSLR KQDFQIR LASVNSPSVEFEIGGK LGAVNLLLR LGVDVAGGADVK LLYSPLQPNIEQGK LQVVAETYENQASIVGNWVTK NPNFASPLLFLDVLLPK NVDEDEDNYAGEFK QKTDVHYR SDGPIEFEISIGNHGNK SLNGEGNFNWR SVLAADQSGLSDPFVR TAWTESHNEAK TPTSPWK VAEPAYTDAQGELK VDAGGAVDVGLR VFVLEDFER VLLELSTELLDEPTADLVKPLEQDK VVVWFGLEK YLPNTLNPEFGK

**Actin-2 [Sm]** EITALAPSTMK DLYANTVLSGGTTMFPGIADR LDLAGRDLTDYLMK DLTDYLMK TTGIVLDSGDGVTHTVPIYEGYALPHAILR VAPEEHPVLLTEAPLNPK SYELPDGQVITIGNER GYSFTTTAER YPIEHGIVTNWDDMEK IVAPPERK IVAPPER IKIVAPPER IWHHTFYNELR AGFAGDDAPR DSYVGDEAQSKR DSYVGDEAQSK RGILTLK HQGVMVGMGQK AVFPSIVGRPR

**Alpha tubulin [Sm]** LDHKFDLMYAK LIGQIVSSITASLR IHFPLATYAPVISAEK LSVDYGKK QLFHPEQLITGK NLDIERPTYTNLNR QLFHPEQLITGKEDAANNYAR VGINYQPPTVVPGGDLAK AFVHWYVGEGMEEGEFSEAR *TIGGGDDSFNTFFSETGAGK FDGALNVDLTEFQTNLVPYPR YEEGEGEGEGDVSDVGVEEYDK EIVDLVLDR

**Tubulin beta chain [Sm]** NSSYFVEWIPNNVK GHYTEGAELVDSVLDVVR FWEVISDEHGIDPTGTYHGDSDLQLER LHFFMPGFAPLTSR GHYTEGAELVDSVLDVVRK *AGPFGQLFRPDNFVFGQSGAGNNWAK IREEYPDR AILVDLEPGTMDSVR YLTVAAIFR LAVNMVPFPR IMNTFSVVPSPK SLTVPELTQQMFDAK

**Tubulin subunit beta [Sm]** GHYTEGAELVDSVLDVVR FWEVISDEHGIDPTGTYHGDSDLQLER LHFFMPGFAPLTSR GHYTEGAELVDSVLDVVRK *AGPFGQLFRPDNFVFGQSGAGNNWAK IREEYPDR AILVDLEPGTMDSVR YLTVAAIFR LAVNMVPFPR IMNTFSVVPSPK SLTVPELTQQMFDAK NSSYFVDWIPNNVK

**Collagen alpha-1(V) chain [Sm]** DGPDGPAGRHGIGPEGK GIPGSPGREGK GTPGPLGMEGK EGDPGIEGESGVNGK LGPDGRQGK EGPIGAGPQGPLGKEGKPGPYGR DGRPGPEGKPGR EGKTGPLGILGDEGLRGTDGR LGRPAPEGIMGKQGPK DGSVGTQGKDGVRGPLGR EGLIGKPGKQGPLGR

**Prominin (prom) protein, putative [Sm]** AWNDLAQSPK ELMELANDQLK FLSVVDIR IDVVSLFK IIDDGLVSFEEDFTK ISLEINEEINDHYLR KIDLESPFR KMDQYVFESLK NELDAIRPTLDIIR SIFYAVEGSLSDSSK VKLQDNALEMLERCEIIK

**Rab GDP-dissociation inhibitor [Sm]** APAIEVISK LLVHTGVTR DWNVDLIPK SPYLYPLYGLGELPQSFAR FLMADGK NALSSQIIIPQNQVGR LSAVYGGTYMLEKPVDEIVIEDGK

**Actin [Sm]** EITALAPSTMK *QEYDESGPGIVHR AVFPSIVGRPR IWHHTFYNELR VAPEEHPVLLTEAPLNPK AGFAGDDAPR

**Synaptotagmin, putative [Sm]** DIDHGEYAGNIIER QLNLDDYLPK QWEEIIK GIYVDEIPGLAHNIEQYK *TNIISNTNRPVWK QHELDILIILK

**Fimbrin [Sm]** RDDFLVEGETLEER *GLIDTITLNR LLHEVTPGESHIPR DIWLGNNIDDASIR LGHDVELQDGILPVDPSVDGQLYQR

**Signal recognition particle 68 kD protein [Sm]** SPASAKLGIK SLADPSPGPAICEEAK QYDGHRLGHQQQASK ESKVPISIGLWDVESVQSAQK DGTVSPESAIATR

**Gelsolin [Sm]** TVELDTVLEEQAVQHR LLHFLQEGGR TAVLDELFTNSSDEFLQYLPDKPVYR TTPNEYQPR

**Intermediate filament proteins [Sm]** LNRSEEVSENLSRELQDCR NGSYIEILNK QDSSQRNEFNRLQEENSK QSAEEALTRLHK

**Cytoplasmic dynein light chain [Sm]** NFGSYVTHETK DIAAFIK DIAAFIKK

**Collagen alpha chain type IV [Sm]** *RTIFCQEIADK AGKFGKLGTLGR

**Microtubule-associated protein 9 [Sm]** KNEKINR *VASLENLVISIDHSNK

**Rab-2,4,14 [Sm]** TAANVEDAFIDTAK *FQPVHDLTIGVEFGAR

**Rab11, putative [Sm]** STIGVEFATK *VVLTGDSGVGK

**Ran [Sm]** HITGEFEKK HITGEFEK

**Tegumental antigen**

**200-kDa GPI-anchored surface glycoprotein [Sm]** AYHQLDENNVDSQLK GILPMDSDEAAESLSK TASPGQLDWMLPSGEK VIGEVDEYPDYIK VTIPETNDDSDEILEK QGDDNDNLSILK IENLLPGR VTVTVSDLEVDINSK DSHLLDIR IYDLIIR QVNYVPITMK LNNPSTSDVYK QTFSLK RSRSSDK SDDDNDDQPYTIK TIHGQIIQFK LHVYGLPFHYPK IVEFGEK IQLTNQGIINTEFSEIMTELR TGQTDLKAPTSGR SPESSDKPGK TTETLNDKNNHVK TQEAQSGPLWYAMESLIFDPVNK SITMGTSSDIRFSTGR

**Tegumental protein Sm 20.8 [Sm]** KFDEKDQAVQLK AWHIVIVK AIELLTSSK FDEKDQAVQLK YVAENKLDDMMVTK LHPDVVVIYEQLPLDR QWLDITYGK DGTETITVEELK SLFDPK

**Tegument antigen SmA 22.6 [Sm]** AFLEIDADSNEMIDKQELIK LIDPWIAR LSQMEEFIR LPPNIEIIAATMSK VSEIRR EYVDNTSR AFLEIDADSNEMIDK

**Tegumental antigen [Sm]** STVADDFVIK QNDSSNLYEITIR DSFLDAFFALDTDNREVISLNDLSSYNK

**Sm23, putative [Sm]** IDSEIDALMTGALDKPTK FSQYGDNLHK

**Sm29 [Sm]** IPSVPIANPFR TQCNTDLCNGLTVDNTGK

**Histones**

**histone H3 [Sm]** KLPFQR STELLIR RVTIMPK EIAQDFKTDLR

**histone H4 [Sm]** DAVTYTEHAK VFLENVIR ISGLIYEETR KTVTAMDVVYALK

**histone H2B [Sm]** LLLPGELAK QVHPDTGISSK LAHYNKK

**Chaperones**

**Heat shock protein 70 (hsp70)-4, putative [Sm]** IGDASQIFSEPR YANKDDLPNQNIVIGTFK FMTHQVEAAR GGGVEVITNEYSER NAVEEYVYEMR EITETTIGSK SEDVHSVELIGGSSR

**Peptidyl-prolyl cis-trans isomerase [Sm]** KMESLGSTSGKPSK IIFELFNDVPDTTR MESLGSTSGKPSKK KMESLGSTSGKPSKK AFFDIK

**Heat shock protein-HSP20/alpha crystallin family [Sm]** DLLTGLEHGGGAHR GVHGLSYVDDGSGGKR MTDDGVLMLEAPVK WDDDMRR MGSLDVPSTGSVNDFLK

**Chaperonin containing t-complex protein 1 epsilon subunit tcpe [Sm]** MIVDEAKR *GVIVDKDFSHPQMPR AQGQLAFDEYGRPFIILR

**Translation**

**Elongation factor 1-alpha (ef-1-alpha) [Sm]** IGGIGTVPVGR PSDKEHINIVVIGHVDSGK NVAVSEIHR IPLQDVYK FDTQNYK YAWVLDK STNMPWFK

**Eukaryotic translation elongation factor [Sm]** GFNQFILSPIYK VFSGTIGTGQK VAVDCVNPADLPK MGVTLNEAELSLPDKQR VIENVNVIISEFEVANNPMGDLTLDVAK

**Others**

**SPRY domain containing protein [Sm]** FVQAVENTGWQGFR LHGDIIENYGEAFR TGIGSADNPHPVGK LETPNFK LEDVPVESIR LGWSTNDASLILGTDSK VEVNFGDKPFNFPPK VESPSISDQQEDEVR *LIDIHYSYNGQLLETPNFK TNWGLK

**Cell division control protein 48 aaa family protein (transitional Endoplasmic reticulum atpase), putative [Sm]** LAGESESNLR IVSQLLTLMDGLK HFEEAMR NAPAIIFIDELDAIAPK LAGESESNLRK LDQLIYIPLPDEASR GILLYGPPGTGK

**Band 4.1-like protein [Sm]** EPSTEWIPR LRDTPGPQR MLQQIVELHK FYPPNIDIFK TLSPGGLK MLQQIVELHKLHK

**Centrosomal protein of 135 kDa (Cep135 protein) [Sm]** HTPSTNAK HIELNLGDTR QNSLQYQLEK EQMEKLKK LSEECRNLK EGRLTISQR

**Excision repair helicase ercc-6-related [Sm]** LVAKQCRYDVGEGMDISLSR QCRYDVGEGMDISLSR *LKADVQIQLPAK *GRLDDVWTLRGK QSNAFRETNSVKMER

**Ubiquitin (ribosomal protein L40) [Sm]** ESTLHLVLR TITLEVEPSDTIENVK IQDKEGIPPDQQR EGIPPDQQR

**Ubiquitin-protein ligase BRE1 [Sm]** DLHWALIGK GVDYVVGMR *LLSHDDPLIHVPTTGSIELR NHVTPVESETIK

**Mixed-lineage leukemia 5 mll5 [Sm]** HVISSHR CATYDFDFPLTIESSKPIVR NFSKQPSDSAKVPVSQLR LNTNVSSKPCLVSQEDSPDK

**Basic helix-loop-helix transcription factor, putative [Sm]** AVSYPEIR QANNARERIR *AVSYPEIRLR

**Late embryogenesis abundant protein [Sm]** TKQSSRQNLK HCEKSLLNK *TSSVEYKLR

**Zinc finger protein [Sm]** EDLSSLGIR *QEDRGNTKSNTPNQVSSHGR

**Gata zinc finger domain-containing protein [Sm]** *DNLNNLILKIQK

**Hypothetical/Unnamed**

**hypothetical protein Smp_140590 [Sm]** ETLSEDTHFPPFDR SNPSTEFDALNLTGR YYDGYENIGSPYDTFK SATLSNSK RNDPPVMVPTR EPSSDIANLK ISGTLPPVGKSANDVMGSR NPVINVAVTER

**hypothetical protein [Sm]** GLQLLEEAIK YMIDLDGTENKEK IEEELGTAAK GVLTAVSNVNK LAGHEDVIMPVPAFNVINGGSHAGNK YMIDLDGTENK LAMQEFMILPTGASSFTEAMQIGTEVYHNLK

**hypothetical protein Smp_007640 [Sm]** GYDELVPHYIDVVKEER LITEAIIQMSDILK TMQEALQR AGNQGIPATPR WPDQVNYNIGIIKPK HIIGIEFIER GFNYHQGPEWLWPTGYYLR

**unnamed protein product [Sm]** GHSFTTTAER VAPEEHPVLLTEAPMNPK TTGIVLDSGDGVSHTVPIYEGYALPHAILR TTGIVLDSG VLDSGDGVSH VLLTEAPMNPK

**hypothetical protein Smp_134750 [Sm]** RPQSNHVTSSLISRQK *TSERIISAQK *TPTYTPLNLSDEK FSDSKLQDLTDRAK *STPISHRPLSSQRIR

**hypothetical protein Smp_080920.3 [Sm]** HLHITDHQGTPYGSSSPDFQLSPEFVLQLK REPTSFISR SLLNDNKETSFTFMNEK

**hypothetical protein Smp_133590 [Sm]** LRSLLTPK *STCRDITAFYNSTAK *GLSNGNICHLTNHQK

**hypothetical protein Smp_024220 [Sm]** SELGLGFADSLR GSLGPSPGLR

**hypothetical protein Smp_155620 [Sm]** *IHYSTENTENIHQPTR LIYPGDQLIK

**hypothetical protein Smp_145450 [Sm]** AAELALR *RKQTQEQIQNDER

**hypothetical protein [Sm]** *AVSRMSSMAK *AVSRMSSMAKLK

**hypothetical protein Smp_159020 [Sm]** SQNNDDNKLIYCSSNK *HHNPSPSNSEKAWHVLR

**hypothetical protein Smp_006830.1 [Sm]** STVQSLDADQLIK GIIHRKLHK

**Host proteins (Mus muscuslus)**

**importin-8 isoform X3 [Mm]** ETENDDVTNVIQK IIVSDHVEFPVR TYAVGIQQVLLK DNIVEGIIR

**keratin, type II cytoskeletal 1b isoform X1 [Mm]** NKYEDEINKR FLEQQNQVLQTK FLEQQNQVL

**keratin, type II cytoskeletal 79 isoform X1 [Mm]** NKYEDEINK NLDLDSIIAEVK NKYED

**keratin Kb40 isoform X2 [Mm]** SLNNQFASFIDK *SLNNQFASFIDKVR

**keratin, type I cytoskeletal 18 isoform X1 [Mm]** LAADDFR SLNNQFASFIDK

**axin-1 isoform X1 [Mm]** *TAVLGGTASGHGK *KLEPCDSNEEKR

**39S ribosomal protein L21 [Mm]** *VNELIATGQYGR *KIIVNPQTILR

**Table S3: Summary of RNA sequencing results**

| # Reads | exosomes | whole worms |
| --- | --- | --- |
| Total (“raw”) reads | 10,953,926 | 12,462,586 |
| Mappable reads^a^ | 8,639,784 | 10,232,965 |
| Reads mapped to known miRNAs^b^ | 74,223 | 236,809 |
| Reads mapped to putative miRNAs^c^ | 4,418 | 13,186 |
| Reads mapped to other RNAs (mRNA, Rfam or Repbase)^d^ | 5,450,691 | 7,979,750 |
| Unmapped reads (“no hits”) | 2,824,993 | 1,600,243 |
| Others^e^ | 285,459 | 402,977 |

^a^ Reads remaining after removal of impurities, low-quality sequences and sequences < 15 nucleotides; ^b^reads aligned with known *S. mansoni* or platyhelminth miRNAs available in miRBase vs. 20;

^c^ reads did not map to known miRNAs but aligned to the *S. mansoni* genome between predicted hairpins;

^d^ reads mapped to *S. mansoni* mRNAs, repetitive sequences in Repbase (http://www.girinst.org/repbase) or other (non-miRNA) small RNA species available in the Rfam database (<http://rfam.janelia.org>);

^e^ reads mapped to the *S. mansoni* genome but are not predicted to form hairpins (disregarded).

**Table S4: miRNA sequences detected in exosomes and whole worms**

| **miR_seq** | **miR_name** | **norm_ EXO_SM** | **norm_W_SM** |
| --- | --- | --- | --- |
| TCCCTGAGACTGATAATTGCT | sma-miR-125b_R-1 | 43,394 | 49,431 |
| TGAGATCGCGATTAAAGCTGGT | sma-bantam | 13,148 | 7,495 |
| TGAAAGACGATGGTAGTGAGAT | sma-miR-71a_R+1 | 12,322 | 12,408 |
| TCCCTGAGACCCTTTGATTGCC | sma-miR-125a | 12,075 | 33,572 |
| CCACCGGGTAGACATTCATTCGC | sma-miR-36-3p | 8,977 | 4,146 |
| AACCCTGTAGACCCGAGTTTGG | sma-miR-10-5p | 3,281 | 19,497 |
| TGACTAGAAAGTGCACTCACTTC | sma-miR-61_R+1 | 3,145 | 7,471 |
| TCACAGCCAGTATTGATGAAC | sma-miR-2a-3p_R-1 | 2,198 | 6,603 |
| TCCCTGAGACCTTAGAGTTGT | sme-lin-4-5p_ 3ss14CA17CG19G- | 1,893 | 5,902 |
| TATTATGCAACGTTTCACTCT | sja-miR-2162-3p | 1,794 | 3,246 |
| TAAATGCATTTTCTGGCCCGTT | sja-miR-277_R+2 | 653 | 1,354 |
| GAGAGATTAAGACTGAACGCC | PC-5p-12974_124 | 580 | 298 |
| TAAATGCATTTTCTGGCCCGT | sja-miR-277_R+1 | 532 | 1,717 |
| ACGGGCTTGGCAGAATTAGCGGGG | PC-3p-8606_176 | 304 | 167 |
| TCCCTGAGACCTTAGAGTTGTCT | PC-5p-1634_720 | 275 | 595 |
| GTCCGGGGTGCAGGCTTC | sme-mir-749-p5 | 275 | 188 |
| GGAGGTAGTTCGTTGTGTGGT | sma-let-7 | 254 | 3,535 |
| TGAAAGACTTGAGTAGTGAGACT | sma-miR-71b-5p_ 1ss23GT | 243 | 254 |
| ACACTGCGAGGCATTGAAT | PC-5p-15294_107 | 207 | 563 |
| GAGATGGATAGTGGCTAGCATTT | PC-3p-8590_176 | 190 | 39 |
| CCTCCGGAATCCCATAGTACT | PC-5p-10382_150 | 160 | 156 |
| TATTGCACTAACCTTCGCCTTG | sma-miR-3479-3p | 140 | 1,545 |
| TGGCGCTTAGTAGAATGTCACCG | PC-5p-14055_115 | 121 | 200 |
| TGATGGATGTAGTATAGG | PC-5p-1776_672 | 117 | 436 |
| ATCCGTGCTGAGATTTCGTCT | sja-miR-3492 | 111 | 42 |
| GTCCCTATCTACTATCTAGCGAAACCACAGCC | PC-5p-33527_52 | 90 | 172 |
| TCCTGGACGCTGGCAAATGCT | PC-3p-15712_105 | 77 | 147 |
| ATCTGTTAGTACTTTGGTCCT | PC-3p-21727_78 | 65 | 26 |
| GAGAGATTAAGACTGAACGCCT | PC-3p-19439_87 | 65 | 16 |
| ATGATGGATGTAGTATAGG | PC-5p-5682_249 | 62 | 119 |
| ACCCGTACCCAATCGTCGAC | PC-3p-46742_38 | 60 | 66 |
| TACCGCTGTCCATAACTATGT | PC-3p-70423_25 | 57 | 43 |
| TGGCGAAGAGTTCTGATTAGT | PC-3p-82189_22 | 54 | 23 |
| TGGAAGACTGGTGATATGTTGTT | sja-miR-7-5p | 50 | 1,093 |
| TGACTAGAAAGTGCACTCACTTCT | sja-miR-61_R+2 | 50 | 283 |
| TCGACGATTGGGTACGGGTCT | PC-3p-49221_36 | 50 | 23 |
| TGATGGATGTAGTATAGGTGGGAGCTGGGTG | PC-3p-36957_48 | 49 | 186 |
| CAGGTCGGGCGTCTAGGCACTT | PC-5p-38154_46 | 48 | 14 |
| CGTACTATGGGATTCCGGAGT | PC-3p-55957_32 | 48 | 4 |
| AGGATACTGGAGCCCATGTGTTT | PC-3p-36544_48 | 45 | 11 |
| ATATCGATGTGTATATGCTCT | PC-3p-45469_39 | 45 | 12 |
| TTCTTAGATTGTAAGCACTCT | PC-5p-58341_30 | 44 | 32 |
| CGTCTCAAGGGACTGTGAAACT | sma-miR-2b-5p_ 1ss22AT | 43 | 38 |
| CTCTTAGCAGGATTGGTGTCT | PC-3p-72151_25 | 43 | 6 |
| TATTGGCAAACAATCTGACT | PC-3p-14824_110 | 40 | 54 |
| CATTTCAGATATACTCTGCT | PC-3p-26877_64 | 40 | 25 |
| AACCGATGTTAGGACGTTTGT | PC-3p-25083_69 | 39 | 9 |
| CATACAACCGACTGGCTTTCC | sma-let-7-p3 | 38 | 132 |
| TGGACGGGGCAATATCTTAAT | PC-3p-126893_14 | 38 | 19 |
| TTCCGATTCGGCATTTCTGATT | PC-5p-61230_29 | 37 | 9 |
| ACTGAGACGGGTGTGGATACT | PC-5p-54721_32 | 37 | 4 |
| AGGCCAATTGAACTACCCTTT | PC-5p-37607_47 | 35 | 20 |
| TGAAAGACGATGGTAGTGAGATAT | egr-miR-71_R+3 | 34 | 16 |
| TGTCCGCGGAACAGTGTACC | PC-5p-54980_32 | 33 | 51 |
| TTTGGTCCCCTTCAACCAGCTGT | sme-miR-133a-3p_ L+1_1ss11GT | 33 | 37 |
| TGATATGTATGGGTTACTTGGT | sma-miR-190-5p_R-1 | 31 | 1,659 |
| TGAAAGACGATGGTAGTGAGATA | egr-miR-71_R+2 | 31 | 64 |
| TCCGCGGACATACGCCGACT | PC-5p-89649_20 | 30 | 21 |
| AGAGGTAGTGATTCAAAAAGTT | sme-let-7d | 30 | 7 |
| TCGAGACGGTTACTAGACACT | PC-5p-109992_16 | 28 | 4 |
| TCCCTTGTTCGACTGTGATGTTT | sma-miR-2c-5p_ R+1_1ss22GT | 27 | 21 |
| GATTTGTCTGACGTTATGTGT | PC-5p-66976_27 | 27 | 6 |
| ATAATCAGACTGAATATGTGT | PC-5p-108368_16 | 26 | 26 |
| TTAGGATCCAAGTATAGCGTTT | PC-3p-84103_21 | 26 | 10 |
| TCCGTGCTGAGATTTCGTCA | sma-miR-3492_L-1 | 24 | 26 |
| TCGACAGATGTTAGTGACTGT | PC-3p-73847_24 | 24 | 8 |
| CCGATGTTAGGACGTTTGTCT | PC-3p-148935_12 | 23 | 8 |
| TCACAGGAATCGAAATACTCT | PC-3p-124334_14 | 23 | 11 |
| TTTCGGCAGTTCAGTGGTACT | PC-3p-68130_26 | 23 | 4 |
| CACTCGTCGCACTGCATGCCT | PC-3p-30972_56 | 21 | 92 |
| GAGAAGCCTGATGGTCGGAGT | PC-3p-87239_20 | 21 | 4 |
| TGTCATGGAGTTGCTCTCTA | PC-3p-22144_77 | 20 | 168 |
| TCGGGCAGTACATTTCTGATT | PC-5p-140928_12 | 20 | 11 |
| CAGAGAATTCATGCAGACTAT | PC-5p-122898_14 | 20 | 5 |
| TCCGGGCATTGGTATAACAGT | PC-3p-124567_14 | 20 | 4 |
| GAGGGCTAGGTCATCTGCGATT | PC-5p-100544_18 | 20 | 3 |
| ATAAACGATTGTCTGAACATT | PC-5p-83501_21 | 18 | 6 |
| CAGACAGAAGGTGTAGCTATT | PC-5p-128535_14 | 18 | 1 |
| TAATACTGTTAGGTAAAGATGCC | sma-miR-8-3p | 17 | 2,404 |
| TGCTGCCTGATAAGAGCTGTGA | egr-miR-745_1ss10GA | 17 | 187 |
| TGATTGTCCATTCGCATTTCTT | sja-miR-219-5p_R+1 | 17 | 161 |
| ACGCCGACATAAACCGTGAAT | PC-3p-114367_15 | 17 | 18 |
| TTAACAATCTGGTAGATGTCT | PC-3p-87348_20 | 17 | 17 |
| CAGACCGTGGACGCCAGTTCT | PC-5p-93611_19 | 17 | 16 |
| CCAATGGTGCACATGGGCTCTT | PC-5p-59222_30 | 17 | 13 |
| GACCCGATTGAGATACGCGCT | PC-5p-108611_16 | 17 | 11 |
| CAACTCGGAGAGGCCAGGTTTTT | PC-5p-130301_13 | 17 | 6 |
| CACGTGTTCCGAGTCTTCTCT | PC-5p-131361_13 | 17 | 1 |
| AAGAAGACCCTGTTGAGCTTGACTCTAGTCCGACGTTGTG | PC-3p-28386_61 | 17 | 11 |
| CTGTTAACCGAGGCCATCTCT | PC-3p-53629_33 | 16 | 21 |
| TTGGATCCCCAACTTTCGACT | PC-5p-151851_11 | 16 | 10 |
| ACCACAAGGGCTGATGAGACT | sma-mir-3492-p3 _1ss18AG | 16 | 10 |
| TTCCGGAAGTTTTGAGTGATT | PC-3p-167297_10 | 16 | 1 |
| TGATGGATGTAGTATAGGTGGGAGCTGGGT | PC-3p-51268_35 | 15 | 86 |
| TGGATGCATATGGCTATGTCT | PC-3p-57721_31 | 14 | 15 |
| AGAGATCCCGCTAGAAGCTTT | PC-3p-54871_32 | 14 | 8 |
| AGGAAGCCCACTGTCGACATT | PC-3p-101048_17 | 14 | 8 |
| TATACCGAGGCTTGAGATGG | PC-5p-149581_12 | 14 | 6 |
| AATAGTCTGCATGAATTCTCT | PC-5p-191011_9 | 14 | 5 |
| ACCCCAAGGAGTCTAGTGAATT | PC-3p-165038_10 | 14 | 4 |
| AGATCCAGAGAACATTGAAGT | PC-5p-123862_14 | 14 | 3 |
| CTCCGGAGATGTTTTGGGGCT | PC-5p-201291_8 | 14 | 2 |
| AGCTCAGTGGTCTATCGGTTATT | PC-5p-26623_65 | 14 | 11 |
| AAAAAGTCCCTGTTCGAGCT | PC-3p-27975_62 | 13 | 51 |
| ACTTCGGCTGGTGATTTCCC | PC-3p-129633_13 | 13 | 10 |
| TAAGGCACGCGGTGAATGTCA | sma-miR-124-3p | 13 | 260 |
| ACGACGTATTCCGAGGTCTCT | PC-5p-47560_37 | 13 | 30 |
| TGAAGAATGTTGGAAAATCT | PC-5p-47385_37 | 13 | 20 |
| CCGAGCTATCGCACTGTTGAT | PC-5p-272816_6 | 13 | 13 |
| TGGAAGACTTGTGATTTAGTTGTT | PC-5p-3990_337 | 11 | 399 |
| GTTTGAGTCTTGATGTG | PC-3p-79807_22 | 11 | 33 |
| ATCGGTATTAATGTTGGTGCTT | PC-3p-146279_12 | 11 | 11 |
| TACCGTCACTTTGGCTGTACT | PC-3p-46989_38 | 10 | 22 |
| CAGCCAAAGTGACTCAGGTATGC | sma-mir-125a-p3 | 10 | 18 |
| AGTGCTCTGAATGTC | egr-mir-219-p5 _1ss11TA | 10 | 16 |
| TGAGTAGAGTGAATTATGACT | PC-5p-108973_16 | 10 | 12 |
| ACTGGTAGGTCCTGGGTTCTTT | PC-3p-47447_37 | 10 | 33 |
| TCCCTGAGACCCTTTGATTGT | sja-miR-125a_R-1 | 9 | 23 |
| ACCAGGGCATGACCAGACTCT | PC-5p-95204_19 | 9 | 16 |
| TATCACAGCCCTGCTTGGGACACT | sma-miR-2b-3p _1ss24AT | 7 | 699 |
| TGTGGGTCTCTTTCTTGTCCATT | sma-mir-61-p5 _1ss23GT | 7 | 10 |
| AAAACATTGTCGATTTCTCC | PC-3p-91555_19 | 7 | 10 |
| TATCACAGTCCAAGCTTTGGT | sma-miR-2e-3p | 6 | 159 |
| TGCTGCCTGATAAGAGCTGTGAT | PC-3p-49410_36 | 6 | 69 |
| TTGCACGAATCTAACGCCTGT | PC-3p-52431_34 | 6 | 12 |
| GAAGATTCAGACAAACAATAGC | PC-3p-88291_20 | 6 | 11 |
| TTTTGCTGGCCTGGTATGAATT | PC-3p-97828_18 | 6 | 10 |
| GTAGACATGTGACCT | sme-mir-2150-p3 _1ss5TA | 4 | 61 |
| GAACCCAGTGGTCTATCGGTTAAGTGCT | PC-5p-64128_28 | 4 | 18 |
| TTTGAGTCCCGAATCTTGCGT | PC-5p-112939_16 | 4 | 17 |
| TTGCTGAGATCGCCTTTTTGT | PC-3p-99572_18 | 4 | 11 |
| GATTCGGCTCAGTGGTCTATCGGTTAAGTGCT | PC-5p-67365_26 | 4 | 24 |
| AAGTCCCTGTTCGAGCTGATT | PC-3p-115660_15 | 4 | 12 |
| TCACAACCTACTTGATTGAGGGG | sja-miR-307_R+3 | 3 | 371 |
| AAATTCGAGTCTATAAGGA | sma-miR-10-3p_R-4 | 3 | 55 |
| CCAGTGACCAGACATATCCCT | sma-miR-190-3p_L+1 | 3 | 44 |
| TTCAACCATGCCATTAGCTGT | PC-5p-95371_19 | 3 | 18 |
| TCACAGCCAATATTGATACCC | PC-3p-118142_15 | 1 | 30 |
| TCTCGCTTCCCCGCCTTTCCCG | sma-mir-71a-p3 | 1 | 15 |
| TACCAACTTTGACTGAGTTATACTGCTTTTGT | sma-miR-2e-5p_R+11 | 1 | 13 |
| TAACTCAGTGGTCTATCGGTTAAGTGCT | PC-5p-86492_21 | 1 | 13 |
| GCTCCGGTAGCTTAGTTGGT | sja-miR-3488 | 1 | 18 |
| GAGTTCAGTGGTCTATCGGTTAAGTGCT | PC-5p-106009_17 | 1 | 10 |
| TAGCCGTGGAGCTGTTCATCGCATTT | PC-5p-4596_298 | 0 | 31 |
| GATTGAGCTGTACTGCTTGGGCT | PC-5p-152299_11 | 0 | 24 |
| TTGGTCCCTATCAACCAGCTAT | sja-miR-133_1ss21GA | 0 | 20 |
| CAACGTCATCCTCATAGTGATT | PC-5p-82254_22 | 0 | 20 |
| TATCACAGCCCTGCTTGGGACACTT | egr-miR-2b_R+3 | 0 | 18 |
| NGGAATGTGGCGAAGTATGGTC | sja-miR-1_1ss1TN | 0 | 17 |
| ATAACTCAGTGGTCTATCGGTTAAGTGCT | PC-5p-78919_23 | 0 | 16 |
| GAAGAGTGCCGACCCGGTTAGCC | sma-mir-36-p5 | 0 | 16 |
| TTGACGTAATGATTTGAG | PC-3p-110194_16 | 0 | 15 |
| GTCATCCTTGGATTGTGATTTT | sma-mir-2d-p5 | 0 | 14 |
| TATCACAGTCCTGCTTAGGTGACG | sma-miR-2d-3p_R+2 | 0 | 13 |
| GTGAGCAAAGTTTCAGGTGT | egr-miR-87-3p | 0 | 13 |
| TTGGTCCCTATCAACCAGCT | sja-mir-133-p5 | 0 | 12 |
| AGGGTTATCCGTTTGCTCCGTT | PC-5p-191498_9 | 0 | 11 |
| TGTAGGGCGGGATTGAGC | PC-5p-127121_14 | 0 | 10 |

| **Table S5: Comparison of protein content with published proteomics  analysis of extracellular vesicles from adult and schistosomule *S. mansoni*.** | | | | | | |
| --- | --- | --- | --- | --- | --- | --- |
| **Metabolic Enzymes** | | | | | | |
|  |  |  |  |  |  |  |
|  |  |  | **Adults** | | **Somules** |  |
| **GI (genInfo identifier)** | **Accession number** | **Name** | **Samoil  *et al*.** | **Sotillo  *et al*.** | **Nowacki  *et al*.** | **ES  protein*** |
| 360045358 | CCD82906.1 | Glycogen phosphorylase [Sm] | Yes | No | No | Yes |
| 391358187 | P16641.3 | Taurocyamine kinase [Sm] | Yes | No | Yes | Yes |
| 156118911 | ABU49845.1 | Creatine kinase [Sm] | Yes | No | Yes | Yes |
| 3023710 | Q27877.1 | Enolase [Sm] | Yes | Yes | Yes | Yes |
| 353230309 | CCD76480.1 | Pyruvate kinase [Sm] | Yes | No | No | Yes |
| 360043735 | CCD81281.1 | Glucose-6-phosphate isomerase [Sm] | Yes | Yes | No | Yes |
| 360045088 | CCD82636.1 | Lactate dehydrogenase [Sm] | Yes | Yes | No | Yes |
| 353228833 | CCD75004.1 | Malate dehydrogenase [Sm] | Yes | No | No | Yes |
| 353229703 | CCD75874.1 | Phosphoenolpyruvate carboxykinase [Sm] | Yes | No | No | Yes |
| 256070409 | XP_002571535.1 | Ornithine-oxo-acid transaminase [Sm] | Yes | No | No | Yes |
| 1172460 | P41759.1 | Phosphoglycerate kinase [Sm] | Yes | No | Yes | Yes |
| 353230092 | CCD76263.1 | Transketolase [Sm] | Yes | Yes | No | Yes |
| 256090542 | XP_002581246.1 | Phosphoglucomutase [Sm] | Yes | No | No | Yes |
| 353232336 | CCD79691.1 | Fructose 1,6-bisphosphate aldolase [Sm] | Yes | Yes | Yes | Yes |
| 350645988 | CCD59265.1 | Aldehyde dehydrogenase, putative [Sm] | Yes | No | No | Yes |
| 350645892 | CCD59437.1 | Adenosylhomocysteinase, putative [Sm] | Yes | No | No | No |
| 353229457 | CCD75628.1 | Glyceraldehyde-3-phosphate dehydrogenase [Sm] | Yes | Yes | Yes | Yes |
| 123502 | P09383.1 | Hypoxanthine-guanine phosphoribosyltransferase [Sm] | Yes | No | No | No |
| 383930643 | AFH56663.1 | Methylthioadenosine phosphorylase [Sm] | Yes | No | No | Yes |
| 256082671 | XP_002577577.1 | Aconitate hydratase [Sm] | Yes | No | No | Yes |
| 3122305 | Q27778.1 | 6-phosphofructokinase [Sm] | Yes | No | No | Yes |
| 360044093 | CCD81640.1 | Long-chain-fatty-acid-CoA ligase [Sm] | Yes | No | No | No |
| 353229440 | CCD75611.1 | Glycogenin-related [Sm] | Yes | No | Yes | No |
| 350643977 | CCD58348.1 | 6-phosphogluconate dehydrogenase,putative [Sm] | Yes | No | No | No |
| **Xenobiotic / Redox Metabolism** | | | | | | |
|  |  |  |  |  |  |  |
| 121700 | P09792.1 | Glutathione S-transferase 28 kDa isozyme [Sm] | Yes | No | Yes | Yes |
| 256093080 | XP_002582203.1 | Glutathione S-transferase 26 kDa [Sm] | Yes | Yes | Yes | Yes |
| 350645579 | CCD59704.1 | Aldo-keto reductase, putative [Sm] | Yes | No | No | Yes |
| 161007 | AAA29889.1 | Glutathione S-transferase, partial [Sm] | Yes | No | No | Yes |
| 353231561 | CCD77979.1 | Glyoxalase I [Sm] | Yes | No | No | No |
| **Proteases** | | | | | | |
|  |  |  |  |  |  |  |
| 350646132 | CCD59179.1 | Thimet oligopeptidase (M03 family) [Sm] | Yes | No | No | No |
| 360043297 | CCD78710.1 | Leucine aminopeptidase (M17 family) [Sm] | Yes | Yes | No | Yes |
| 353230291 | CCD76462.1 | Calpain (C02 family) [Sm] | Yes | Yes | Yes | Yes |
| 729709 | P09841.3 | Hemoglobinase (Antigen SM32) [Sm] | Yes | No | No | No |
| 353228442 | CCD74613.1 | Cathepsin B-like peptidase (C01 family) [Sm] | Yes | No | No | Yes |
| 256072593 | XP_002572619.1 | Prolyl oligopeptidase (S09 family) [Sm] | Yes | No | No | No |
| 353230839 | CCD77256.1 | SpAN g.p. (M12 family) [Sm] | Yes | No | No | No |
| 564131894 | AHB79081.1 | Serine protease 2 precursor [Sm] | Yes | Yes | No | Yes |
| 256084114 | XP_002578277.1 | Subfamily M12B unassigned peptidase (M12 family) [Sm] | Yes | No | No | No |
| 353233303 | CCD80658.1 | Family S9 non-peptidase homologue (S09 family) [Sm] | Yes | No | No | No |
| 353232118 | CCD79473.1 | Xaa-Pro dipeptidase (M24 family) [Sm] | Yes | No | No | No |
| **Fatty acid binding** | | | | | | |
|  |  |  |  |  |  |  |
| 55670478 | 1VYG | Fatty Acid Binding Protein [Sm] | Yes | No | Yes | Yes |
| **Transporters/channels** | | | | | | |
|  |  |  |  |  |  |  |
| 350644272 | CCD60986.1 | Plasma membrane calcium-transporting atpase, putative [Sm] | Yes | No | No | No |
| 360043551 | CCD78964.1 | Sodium potassium transporting ATPase alpha subunit [Sm] | Yes | No | No | No |
| 353231052 | CCD77470.1 | Glucose transport protein [Sm] | Yes | No | No | No |
| 353233037 | CCD80392.1 | Cation-transporting ATPase [Sm] | Yes | No | No | No |
| 256084157 | XP_002578298 | Choline transporter-like protein 2 (Ctl2) [Sm] | Yes | No | No | No |
| 353229720 | CCD75891.1 | Aquaporin-3 [Sm] | Yes | No | No | No |
| 256084334 | XP_002578385.1 | Chloride channel protein [Sm] | Yes | No | No | No |
| 353231352 | CCD77770.1 | Anion exchange protein [Sm] | Yes | No | No | No |
| **Signal Transduction and Biological Regulation** | | | | | | |
|  |  |  |  |  |  |  |
| 3023193 | Q26540.1 | 14-3-3 protein homolog 1 [Sm] | Yes | Yes | Yes | Yes |
| 353228883 | CCD75054.1 | 14-3-3 epsilon [Sm] | Yes | No | Yes | Yes |
| 256084742 | XP_002578585.1 | Annexin [Sm] | Yes | Yes | Yes | Yes |
| 353228653 | CCD74824.1 | sh3 domain grb2-like protein B1 (endophilin B1) [Sm] | Yes | No | No | No |
| 353228669 | CCD74840.1 | Kynurenine aminotransferase [Sm] | Yes | No | No | No |
| 353231089 | CCD77507.1 | Talin [Sm] | Yes | No | No | No |
| 256082840 | XP_002577660.1 | Integrin alpha-ps [Sm] | Yes | No | No | No |
| 353228490 | CCD74661.1 | Integrin beta subunit [Sm] | Yes | No | No | No |
| 353232319 | CCD79674.1 | Atp-diphosphohydrolase 1 [Sm] | Yes | No | No | Yes |
| 256084780 | XP_002578604.1 | rpgr-interacting protein 1 related [Sm] | Yes | No | No | No |
| 350646699 | CCD58613.1 | Hyperpolarization activated cyclic nucleotide-gated potassium channel, putative [Sm] | Yes | No | No | No |
| 256080579 | XP_002576557.1 | ng, ng-dimethylarginine dimethylaminohydrolase [Sm] | Yes | Yes | No | No |
| 256052204 | XP_002569666.1 | ral guanine nucleotide dissociation stimulator ralgds[Sm] | Yes | No | No | No |
| 353229530 | CCD75701.1 | Proline-serine-threonine phosphatase interacting protein [Sm] | Yes | No | No | No |
| 360043093 | CCD78505.1 | Lip-related protein (liprin) alpha [Sm] | Yes | No | No | No |
| 256090851 | XP_002581393.1 | Tetraspanin [Sm] | Yes | No | Yes | No |
| 256089413 | XP_002580804.1 | Serine/threonine protein kinase [Sm] | Yes | No | No | No |
| 360043400 | CCD78813.1 | rap1 [Sm] | Yes | Yes | Yes | No |
| 350646635 | CCD58662.1 | Calponin homolog, putative [Sm] | Yes | No | No | Yes |
| 360042647 | CCD78057.1 | Voltage-gated potassium channel [Sm] | Yes | No | No | No |
| 353233207 | CCD80562.1 | Syntenin [Sm] | Yes | Yes | No | No |
| **Cytoskeletal /Structural** | | | | | | |
|  |  |  |  |  |  |  |
| 350646584 | CCD58796.1 | Fer-1-related [Sm] | Yes | No | No | No |
| 1703114 | P53471.1 | Actin-2 [Sm] | Yes | Yes | Yes | Yes |
| 353232589 | CCD79944.1 | Alpha tubulin [Sm] | Yes | No | No | Yes |
| 353232516 | CCD79871.1 | Tubulin beta chain [Sm] | Yes | No | No | Yes |
| 256076432 | XP_002574516.1 | Tubulin subunit beta [Sm] | Yes | No | No | Yes |
| 353229949 | CCD76120.1 | Collagen alpha-1(V) chain [Sm] | Yes | No | No | No |
| 350645253 | CCD60034.1 | Prominin (prom) protein, putative [Sm] | Yes | No | No | No |
| 353231032 | CCD77450.1 | Rab GDP-dissociation inhibitor [Sm] | Yes | No | Yes | No |
| 360045419 | CCD82967.1 | Actin [Sm] | Yes | Yes | Yes | Yes |
| 350644919 | CCD60380.1 | Synaptotagmin, putative [Sm] | Yes | No | No | No |
| 495668 | AAA29882.1 | Fimbrin [Sm] | Yes | No | Yes | Yes |
| 360044904 | CCD82452.1 | Signal recognition particle 68 kD protein [Sm] | Yes | No | No | No |
| 256072032 | XP_002572341.1 | Gelsolin [Sm] | Yes | No | No | No |
| 353230603 | CCD77020.1 | Intermediate filament proteins [Sm] | Yes | No | No | No |
| 353230415 | CCD76586.1 | Cytoplasmic dynein light chain [Sm] | Yes | Yes | No | Yes |
| 256073059 | XP_002572850.1 | Collagen alpha chain type IV [Sm] | Yes | No | No | No |
| 353232499 | CCD79854.1 | Microtubule-associated protein 9 [Sm] | Yes | No | No | No |
| 360045234 | CCD82782.1 | Rab-2,4,14 [Sm] | Yes | No | No | No |
| 350645028 | CCD60258.1 | Rab11, putative [Sm] | Yes | No | Yes | No |
| 353228708 | CCD74879.1 | Ran [Sm] | Yes | No | No | No |
| **Tegumental antigen** | | | | | | |
|  |  |  |  |  |  |  |
| 360043686 | CCD81232.1 | 200-kDa GPI-anchored surface glycoprotein [Sm] | Yes | No | No | No |
| 353230232 | CCD76403.1 | Tegumental protein Sm 20.8 [Sm] | Yes | Yes | Yes | Yes |
| 135578 | P14202.1 | Tegument antigen SmA 22.6 [Sm] | Yes | Yes | No | Yes |
| 390124514 | CCE94318.1 | Tegumental antigen [Sm] | Yes | No | No | No |
| 350646174 | CCD59158.1 | Sm23, putative [Sm] | Yes | No | No | No |
| 353230115 | CCD76286.1 | Sm29 [Sm] | Yes | Yes | Yes | No |
| **Histones** | | | | | | |
|  |  |  |  |  |  |  |
| 353231319 | CCD77737.1 | histone H3 [Sm] | Yes | No | No | Yes |
| 10953803 | AAG25601.1 | histone H4 [Sm] | Yes | Yes | No | Yes |
| 353229586 | CCD75757.1 | histone H2B [Sm] | Yes | No | No | Yes |
| **Chaperones** | | | | | | |
|  |  |  |  |  |  |  |
| 238663484 | CAZ34365.1 | Heat shock protein 70 (hsp70)-4, putative [Sm] | Yes | No | No | Yes |
| 27805450 | Q26565.1 | Peptidyl-prolyl cis-trans isomerase [Sm] | Yes | No | No | Yes |
| 353230032 | CCD76203.1 | Heat shock protein-HSP20/alpha crystallin family [Sm] | Yes | No | No | No |
| 256082744 | XP_002577613.1 | Chaperonin containing t-complex protein 1 epsilon subunit tcpe [Sm] | Yes | No | No | No |
| **Translation** | | | | | | |
|  |  |  |  |  |  |  |
| 353230261 | CCD76432.1 | Elongation factor 1-alpha (ef-1-alpha) [Sm] | Yes | Yes | Yes | Yes |
| 353231791 | CCD79146.1 | Eukaryotic translation elongation factor [Sm] | Yes | No | No | Yes |
| **Others** | | | | | | |
|  |  |  |  |  |  |  |
| 256080932 | XP_002576729.1 | SPRY domain containing protein [Sm] | Yes | No | No | No |
| 350644553 | CCD60716.1 | Cell division control protein 48 aaa family protein (transitional Endoplasmic reticulum atpase), putative [Sm] | Yes | No | No | No |
| 360045009 | CCD82557.1 | Band 4.1-like protein [Sm] | Yes | No | No | No |
| 350646643 | CCD58670.1 | Centrosomal protein of 135 kDa (Cep135 protein) [Sm] | Yes | No | No | No |
| 360044828 | CCD82376.1 | Excision repair helicase ercc-6-related [Sm] | Yes | No | No | No |
| 256079432 | XP_002575991.1 | Ubiquitin (ribosomal protein L40) [Sm] | Yes | No | Yes | Yes |
| 353230536 | CCD76953.1 | Ubiquitin-protein ligase BRE1 [Sm] | Yes | No | No | No |
| 256084236 | XP_002578337.1 | Mixed-lineage leukemia 5 mll5 [Sm] | Yes | No | No | No |
| 350646702 | CCD58616.1 | Basic helix-loop-helix transcription factor, putative [Sm] | Yes | No | No | No |
| 353231449 | CCD77867.1 | Late embryogenesis abundant protein [Sm] | Yes | No | No | No |
| 353232869 | CCD80225.1 | Zinc finger protein [Sm] | Yes | No | No | No |
| **Hypothetical/Unnamed** | | | | | | |
|  |  |  |  |  |  |  |
| 353229181 | CCD75352.1 | hypothetical protein Smp_140590 [Sm] | Yes | No | No | No |
| 256068121 | XP_002570696.1 | hypothetical protein [Sm] | Yes | No | No | No |
| 360043835 | CCD81381.1 | hypothetical protein Smp_007640 [Sm] | Yes | No | No | No |
| 353233031 | CCD80386.1 | unnamed protein product [Sm] | Yes | No | No | No |
| 360043421 | CCD78834.1 | hypothetical protein Smp_134750 [Sm] | Yes | No | No | No |
| 360044459 | CCD82007.1 | hypothetical protein Smp_080920.3 [Sm] | Yes | No | No | No |
| 353231528 | CCD77946.1 | hypothetical protein Smp_133590 [Sm] | Yes | No | No | No |
| 360044961 | CCD82509.1 | hypothetical protein Smp_024220 [Sm] | Yes | No | No | No |
| 350646356 | CCD58986.1 | hypothetical protein Smp_155620 [Sm] | Yes | No | No | No |
| 353232008 | CCD79363.1 | hypothetical protein Smp_145450 [Sm] | Yes | No | No | No |
| 256078659 | XP_002575612.1 | hypothetical protein [Sm] | Yes | No | No | No |
| 353229633 | CCD75804.1 | hypothetical protein Smp_159020 [Sm] | Yes | No | No | No |
| 353229931 | CCD76102.1 | hypothetical protein Smp_006830.1 [Sm] | Yes | No | No | No |
|  |  |  |  |  |  |  |
|  |  |  |  |  |  |  |
|  |  |  |  |  |  |  |
|  |  |  |  |  |  |  |
